# Functional specialization of different PI3K isoforms for the control of neuronal architecture, synaptic plasticity, and cognition

**DOI:** 10.1126/sciadv.abq8109

**Published:** 2022-11-23

**Authors:** Carla Sánchez-Castillo, María I. Cuartero, Alba Fernández-Rodrigo, Víctor Briz, Sergio López-García, Raquel Jiménez-Sánchez, Juan A. López, Mariona Graupera, José A. Esteban

**Affiliations:** ^1^ Department of Molecular Neuropathology, Centro de Biología Molecular Severo Ochoa (CSIC-UAM), Madrid, Spain.; ^2^ Proteomics Unit, Centro Nacional de Investigaciones Cardiovasculares Carlos III (CNIC), Madrid, Spain.; ^3^ Centro de Investigación Biomédica en Red de Enfermedades Cardiovasculares (CIBERCV), Instituto de Salud Carlos III, Madrid, Spain.; ^4^ Endothelial Pathobiology and Microenviroment Group, Josep Carreras Leukaemia Research Institute (IJC), 08916 Badalona, Barcelona, Spain.; ^5^ Centro de Investigación Biomédica en Red de Cáncer (CIBERONC), Instituto de Salud Carlos III, Madrid, Spain.

## Abstract

Neuronal connectivity and activity-dependent synaptic plasticity are fundamental properties that support brain function and cognitive performance. Phosphatidylinositol 3-kinase (PI3K) intracellular signaling controls multiple mechanisms mediating neuronal growth, synaptic structure, and plasticity. However, it is still unclear how these pleiotropic functions are integrated at molecular and cellular levels. To address this issue, we used neuron-specific virally delivered Cre expression to delete either p110α or p110β (the two major catalytic isoforms of type I PI3K) from the hippocampus of adult mice. We found that dendritic and postsynaptic structures are almost exclusively supported by p110α activity, whereas p110β controls neurotransmitter release and metabotropic glutamate receptor–dependent long-term depression at the presynaptic terminal. In addition to these separate functions, p110α and p110β jointly contribute to *N*-methyl-d-aspartate receptor–dependent postsynaptic long-term potentiation. This molecular and functional specialization is reflected in different proteomes controlled by each isoform and in distinct behavioral alterations for learning/memory and sociability in mice lacking p110α or p110β.

## Teaser

Neurons control their growth and plasticity by means of specific PI3K isoforms, acting on different synaptic compartments.

## INTRODUCTION

The experience-dependent modification of synaptic connections, known as synaptic plasticity, is considered the cellular basis for learning and memory (*1*, *2*). The molecular mechanisms mediating these synaptic changes are still being elucidated, but it is becoming increasingly clear that neurons recruit common intracellular trafficking machinery and signaling pathways for these highly specialized processes. In particular, it is intriguing that synaptic plasticity and cellular growth/survival often rely on common signaling drivers (*3*). This is best exemplified by phosphatidylinositol 3-kinase (PI3K)/phosphatase and tensin homolog (PTEN) signaling: While PI3K activity is generally associated with synaptic potentiation and cellular growth, PTEN is linked to synaptic depression and growth restriction (*4*–*11*). However, this relation is far from univocal. Thus, it is well established that signaling through PI3Ks is a key mechanism mediating long-term potentiation (LTP) induced by *N*-methyl-d-aspartate (NMDA) receptors (NMDARs) (*12*–*16*). However, PI3K has also been associated with long-term depression (LTD), most consistently when induced by metabotropic glutamate receptors (mGluRs) (*17*–*19*) and by NMDARs (*20*, *21*). Together with these established roles in synaptic plasticity, the PI3K pathway drives neuronal growth and structural plasticity during synaptogenesis and neuronal development, exerting direct effects on dendritic complexity, spine number, and shape (*22*–*29*).

These pleiotropic (and sometimes antagonistic) effects driven by PI3K activity are possibly related to the molecular diversity of PI3Ks. Class IA PI3Ks are heterodimers consisting of a regulatory subunit (usually p85α or p85β) and a catalytic subunit (p110α, p110β, or p110δ) that catalyzes the production of phosphatidylinositol 3,4,5-trisphosphate (PIP_3_) in response to ligand stimulation (*30*, *31*). Some isoform-specific functions of PI3Ks are starting to be uncovered by means of gene-targeting approaches and from genetic data on human pathologies (*32*–*35*). Nevertheless, at this moment, the specific roles of individual PI3K isoforms in synaptic plasticity and neuronal morphology are largely unknown, as all PI3K isoforms are expressed in the brain, particularly in neurons. This is not only an academic issue. The knowledge of specific PI3K isoforms engaged for distinct neuronal functions will not only help to understand the regulatory mechanisms involved but may also provide more specific targets for certain pathological conditions.

Among class IA PI3K catalytic subunits, p110α and p110β are ubiquitously expressed, and their absence results in embryonic lethality (*36*, *37*). In neurons, these isoforms are well expressed throughout life. Therefore, we hypothesized that p110α and p110β isoforms could have nonredundant roles in mature neurons regarding neuronal architecture, synaptic plasticity, and ultimately cognitive function. To bypass embryonic lethality, we have used a conditional and region-restricted gene-targeting approach, in combination with proteomic, imaging, electrophysiological, and behavioral assays. On the basis of these experiments, we now report that neuronal ablation of p110α or p110β differentially affects protein expression profiles, suggesting that both isoforms have separate functions in neurons. p110α and p110β have different roles in synapse and dendritic maintenance, basal synaptic transmission, and synaptic plasticity. Last, mice lacking p110α or p110β in the adult hippocampus exhibit distinct behavioral phenotypes in memory and social behavior.

## RESULTS

### Proteomic analyses of mice lacking neuronal p110α or p110β evidence differential cellular processes controlled by the two isoforms

As mentioned above, PI3K signaling controls a variety of neuronal functions, including cell survival, neural circuit development, and synaptic plasticity (*12*–*15*, *17*, *26*). To get some insight into potentially different functions of the catalytic isoforms p110α and p110β, we used a gene-targeting approach using Cre-lox recombination. To bypass the embryonic lethality of the complete knockout of *PIK3CA* and *PIK3CB* genes (*36*, *37*), we bilaterally injected adeno-associated viruses (AAVs) expressing Cre recombinase under the calcium/calmodulin-dependent protein kinase IIα (CaMKIIα) promoter (AAV5-CaMKIIα-mCherry-Cre) into the hippocampus of adult p110α^*flox*/*flox*^ and p110β^*flox*/*flox*^ mice (fig. S1A). Four weeks after injection, Cre was expressed mainly in neurons along the whole septotemporal axis of the hippocampus (*38*) with around 90% infection efficiency in dorsal and ventral CA1 neurons (fig. S1, B to E). As expected, the resulting neuronal p110α and p110β knockout mice (p110α^nKO^ and p110β^nKO^, respectively) displayed a selective reduction in the p110α and p110β protein levels of more than 50% (fig. S2, A and B). To note, the knockout efficiency is not expected to be 100% as p110α and p110β are ubiquitously expressed and are expected to be present in glial cells and cerebral vasculature (*39*, *40*). In both cases, downstream targets of the PI3K pathway seemed to be mostly unaltered (except for S6 expression in p110α^nKO^; fig. S2, C to E).

**Fig. 1.**
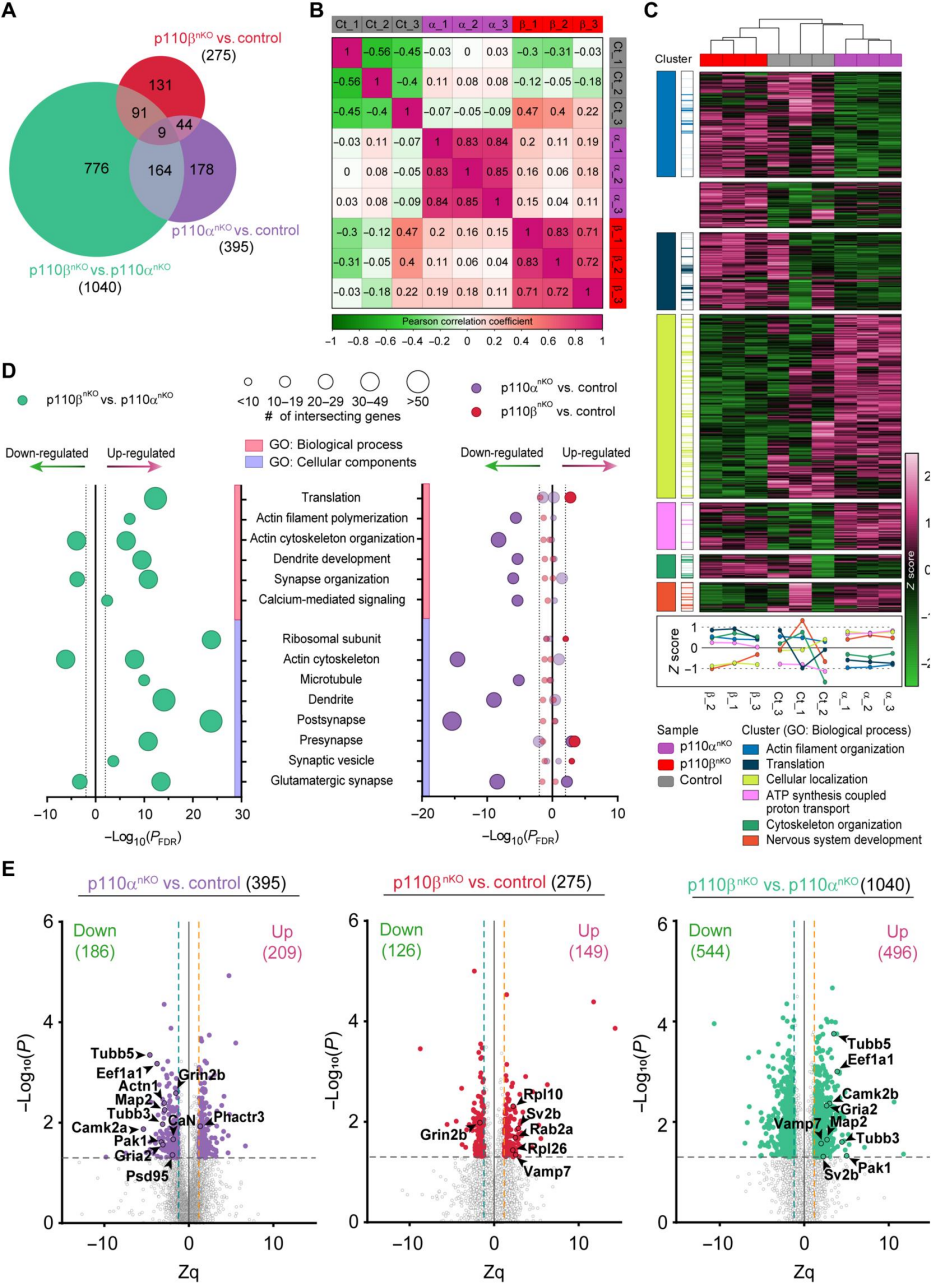
Proteomic analysis of mice lacking neuronal p110α or p110β. (**A**) Venn diagram of DEPs for all possible comparisons. (**B**) Similarity matrix showing pairwise distances between samples, measured by Pearson correlation. (**C**) Unsupervised hierarchical clustering based on Euclidean distances for the 1710 DEPs. Colors represent row-scaled expression values (green, low expression; magenta, high expression). Seven protein clusters were identified on the basis of their expression pattern, and GO biological process enrichment analysis was performed on each cluster. GO biological process terms are annotated for each cluster (left cluster panel), and the intersecting proteins from the dataset are highlighted with the same color code (right cluster panel). Clusters are also represented by sample expression levels in the panel below the heatmap. (**D**) GO enrichment analysis of the DEPs (|Zq| > 1.2 and *P* < 0.05) for p110β versus p110α (left plot) and for p110α and p110β versus control (right plot). For both plots, GO biological process and cell components are plotted against the adjusted *P* value by Benjamini-Hochberg false discovery rate (FDR) correction (*P*_FDR_). Dotted lines represent significance threshold (*P*_FDR_ < 0.01, |log_10_(*P*_FDR_)| > 2) for all GO terms, and the size of the points represents the number of intersecting genes between the dataset and the GO biological process or the GO cell component. Sign on the *x* axis represents up- or down-regulation. *n* = 3 mice per group. (**E**) Volcano plots of DEPs (|Zq| > 1.2 and *P* < 0.05) comparing p110α^nKO^ versus control (left), p110β^nKO^ versus control (center), and p110β^nKO^ versus p110α^nKO^ (right). Each point represents the average fold change of one protein and the *P* value for that comparison. Dashed lines represent significance thresholds [|Zq| > 1.2 and –log_10_(*P*) > 1.3]. Sign on the *x* axis represents up- or down-regulation. Names and outlined points represent top differentially up- or down-regulated proteins.

To start investigating the cellular programs that may be specifically affected by p110α or p110β deficiency, we performed tandem mass tagging (TMT)–based quantitative proteomic analysis to identify hippocampal proteins whose levels were altered in p110α^nKO^ and p110β^nKO^ mice (Fig. 1 and fig. S3). Of the total of 7888 proteins identified, we found 1710 differentially expressed proteins (DEPs) between our three experimental conditions (p110α^nKO^, p110β^nKO^, and wild-type mice infected with the same AAV; three animals per genotype). From these, 1040 DEPs arise from comparing p110β^nKO^ versus p110α^nKO^ proteomic profiles, 395 correspond to p110α^nKO^ versus control, and 275 correspond to p110β^nKO^ versus control (Fig. 1A and data S1). Some of these DEPs are unique to each comparison, and others are common as depicted by the numbers on the intersections of the Venn diagram. Pairwise comparison of DEPs across individual animals (Fig. 1B) showed high correlation coefficients in the replicates from both p110α^nKO^ and p110β^nKO^, indicating high reproducibility among the three replicates (and substantial differences across the three groups). p110α^nKO^, p110β^nKO^, and control replicates clustered together by hierarchical clustering of all DEPs (Fig. 1C). Gene Ontology (GO) enrichment analysis for the proteins comprising each cluster identified as the top GO biological processes: actin filament organization, translation, cellular localization, adenosine 5′-triphosphate (ATP) synthesis–coupled proton transport, cytoskeleton organization, and nervous system development (Fig. 1C). For most of the clusters (except for ATP synthesis), lack of p110α and p110β had opposite effects. The absence of p110α on pyramidal neurons seems to down-regulate actin cytoskeleton and translation-related proteins while promoting an increase in proteins involved in cellular localization and nervous system development. Conversely, deletion of p110β most notably decreased expression of proteins related to cellular localization and neuronal system development.

To further explore the cellular processes and pathways most affected by the absence of p110α and p110β, we performed an enrichment analysis (Fig. 1D). From the comparison of the 1040 DEPs between p110α^nKO^ and p110β^nKO^, we found several categories in both biological and cellular components that exhibited marked changes between PI3K isoforms. Notably, the most remarkable differences were found in actin cytoskeleton and organization and filament polymerization, dendrite development and synapse organization, as well as postsynaptic and dendritic components. In addition, we found several presynaptic proteins (synaptic vesicle glycoprotein 2B [SV2B] and vesicle-associated membrane protein 7 [VAMP7]) up-regulated in p110β^nKO^ and postsynaptic and structural proteins (PSD95, CaMKIIα, AMPA receptor subunit 2 [GluA2], α–actinin-1, and microtubule-associated protein 2 [MAP2]) down-regulated in p110α^nKO^ (Fig. 1E). These results already point out to p110α and p110β as important (and differing) master regulators of synaptic and neuronal structure.

Last, the differential expression profiles caused by neuronal deletion of p110α and p110β were further evaluated by Western blot (fig. S4). We found that AMPA receptor (AMPAR) and NMDAR subunits were significantly decreased in hippocampal lysates from both p110α^nKO^ and p110β^nKO^. The phosphorylation of cofilin was significantly increased solely in the case of p110α^nKO^, reinforcing the link between p110α and the actin cytoskeleton. In addition, the levels of the Ca^2+^/calmodulin-dependent phosphatase calcineurin (CaN) and the phosphorylation of αCaMKII were specifically affected by p110β deletion on pyramidal neurons. Therefore, together, these changes suggest that p110α and p110β isoforms might play differential roles in synaptic function and intracellular signaling.

### Loss of p110α but not p110β impairs dendritic and spine maintenance in hippocampal pyramidal neurons

**Fig. 2.**
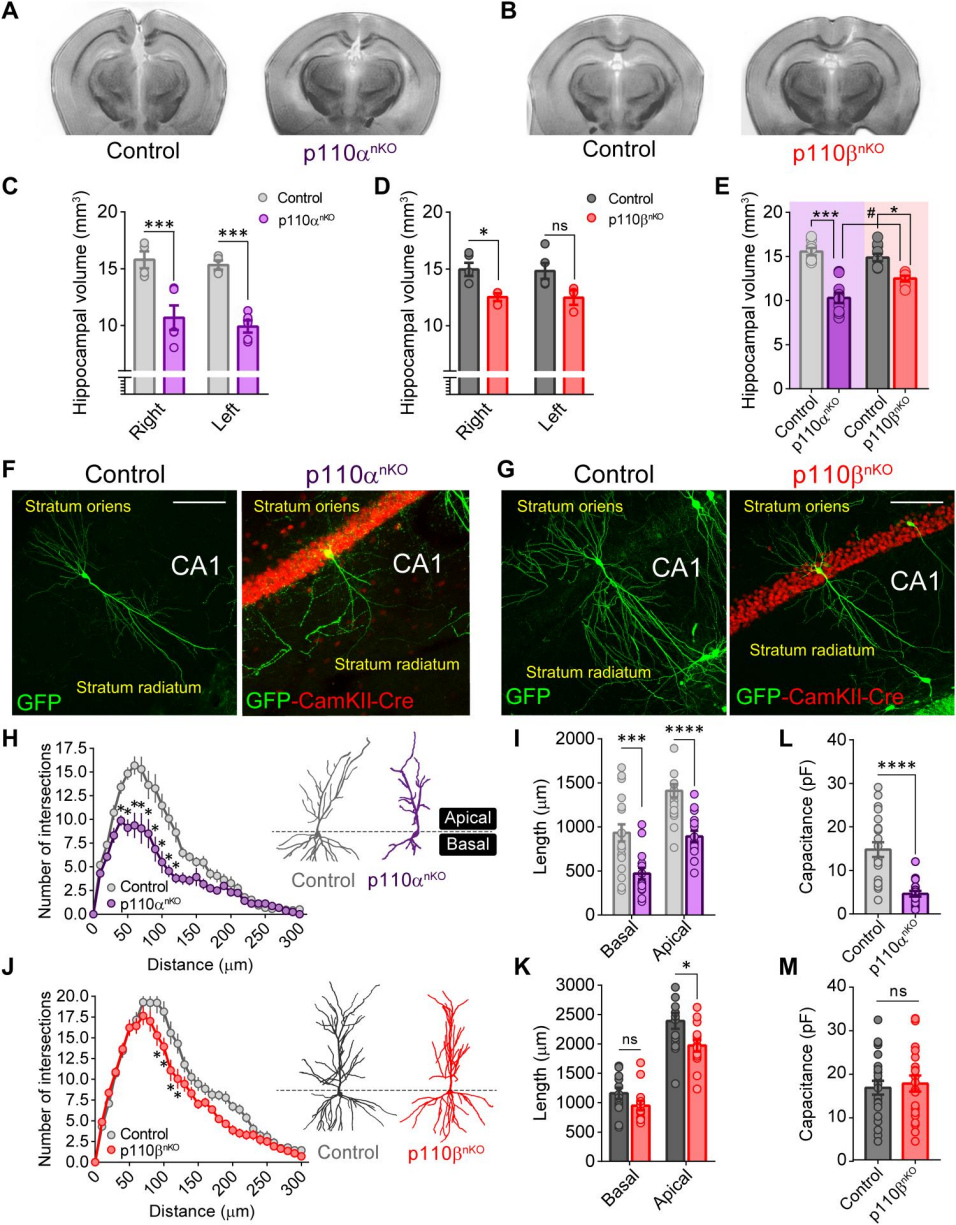
Differential contribution of p110α and p110β to hippocampal size and neuronal morphology. (**A** and **B**) Representative coronal sections of ex vivo magnetic resonance imaging from the brain of p110α^nKO^ and p110β^nKO^ mice after AAV-CaMKII-Cre hippocampal infection. (**C** and **D**) Hippocampal volume quantification for the right and the left hemisphere in p110α^nKO^ and p110β^nKO^ mice and their respective controls. Statistical significance was calculated by two-way repeated-measures analysis of variance (ANOVA) [(C) *F*(1,7) = 30.24, *P* = 0.0009; (D) *F*(1,6) = 8.205, *P* = 0.03] with Bonferroni’s posttest (***P < 0.005 and *P < 0.05). ns, not significant. (**E**) Comparison between the hippocampal volume (right and left hemispheres for each mouse are displayed together) of p110α^nKO^ and p110β^nKO^ mice. Statistical significance was calculated by two-way ANOVA [*F*(1,30) = 8.449, *P* = 0.007] with Tukey’s posttest (***P < 0.005, *P < 0.05, and #*P* < 0.05). p110α*^flox/flox^*: vehicle, *n* = 4; p110α^nKO^, *n* = 5. p110β*^flox/flox^*: vehicle, *n* = 5; p110β^nKO^, *n* = 3. (**F** and **G**) Merged representative images of CA1 green fluorescent protein (GFP)–expressing neurons from p110α*^flox/flox^* and p110β*^flox/flox^* mice for all conditions (saline and AAV injected). After AAV infection, mice were superinfected with a Sindbis GFP virus. Scale bars, 100 μm. (**H** and **J**) Sholl analysis depicting the mean number of intersections along the dendritic tree for uninfected (control) and AAV-infected CA1 pyramidal neurons from p110α^nKO^ and p110β^nKO^ mice. Representative images of neurons traced for each condition are also shown. Statistical significance was calculated by two-way repeated-measures ANOVA [p110α^nKO^: *F*(30,990) = 6.598, *P* < 0.0001; p110β^nKO^: *F*(30,780) = 2.534, *P* < 0.0001] with Bonferroni’s posttest (*P < 0.05). (**I** and **K**) Quantification of the total dendritic length for apical and basal dendrites for p110α^nKO^ and p110β^nKO^ mice. *n* = 14 to 20 neurons from at least three mice per condition. (**L** and **M**) Whole-cell membrane capacitance of AAV-infected (p110α^nKO^ and p110β^nKO^) and uninfected (control) CA1 pyramidal neurons from acute hippocampal slices. Data were compared by Mann-Whitney test (*P < 0.05, ***P < 0.005, and ****P < 0.0001). p110α*^flox/flox^*: vehicle, *n* = 21; p110α^nKO^, *n* = 18 neurons. p110β*^flox/flox^*: vehicle, *n* = 23; p110β^nKO^, *n* = 22 neurons, from four mice per condition. Data are displayed as means ± SEM.

Our previous proteomic data clearly pointed to proteins linked to the actin cytoskeleton, particularly in mice lacking p110α. Therefore, we evaluated whether the absence of p110α or p110β leads to structural or morphological hippocampal alterations. We first analyzed the hippocampal volume of p110α^nKO^ and p110β^nKO^ by ex vivo magnetic resonance imaging approximately 1 month after hippocampal AAV delivery (Fig. 2, A to E, and fig. S5). Both p110α^nKO^ and p110β^nKO^ mice displayed a significant reduction in hippocampal volume (Fig. 2, A to D), although this reduction was significantly more pronounced in p110α^nKO^ as compared to p110β^nKO^ mice (33.8 ± 3.4% of hippocampal volume loss in p110α^nKO^ versus 16.4 ± 2.7% in p110β^nKO^; Fig. 2E). To note, neither the volume of the hemispheres nor the cortex was altered in p110α^nKO^ or p110β^nKO^ mice, supporting the specificity of the hippocampal AAV delivery (fig. S5, C, D, H, and I). These results suggest that the p110α isoform plays a major role in maintaining proper brain growth and size, particularly as compared to p110β.

Previous studies have suggested that the PI3K pathway is important for both dendritic arborization and synaptogenesis (*23*, *26*). Therefore, we hypothesized that the reductions we observed in hippocampal volume could be due to neuronal atrophy. To explore this possibility, p110α^nKO^ and p110β^nKO^ mice were infected for 24 hours with a green fluorescent protein (GFP) Sindbis virus to reveal CA1 neuronal morphology (Fig. 2, F and G). As shown in Fig. 2 (H and I), p110α removal caused a marked decrease in dendritic complexity (as determined by Sholl analysis) and in the total length of both basal and apical dendrites when compared to control mice. A slight decrease in dendritic branching was also found upon p110β deletion in apical dendrites (Fig. 2, J and K), although it was much less pronounced than in p110α-deleted neurons. As a complementary approach, we measured whole-cell membrane capacitance from patch-clamp electrophysiological recordings in acute hippocampal slices as an indicator of total cell surface (Fig. 2, L and M). In agreement with our morphological analysis, whole-cell membrane capacitance was strongly reduced in neurons from p110α^nKO^ mice but showed no differences in p110β^nKO^ neurons when compared to controls. Therefore, these combined results reinforce the notion that p110α activity is specifically required for the maintenance of proper dendritic and neuronal architecture.

**Fig. 3.**
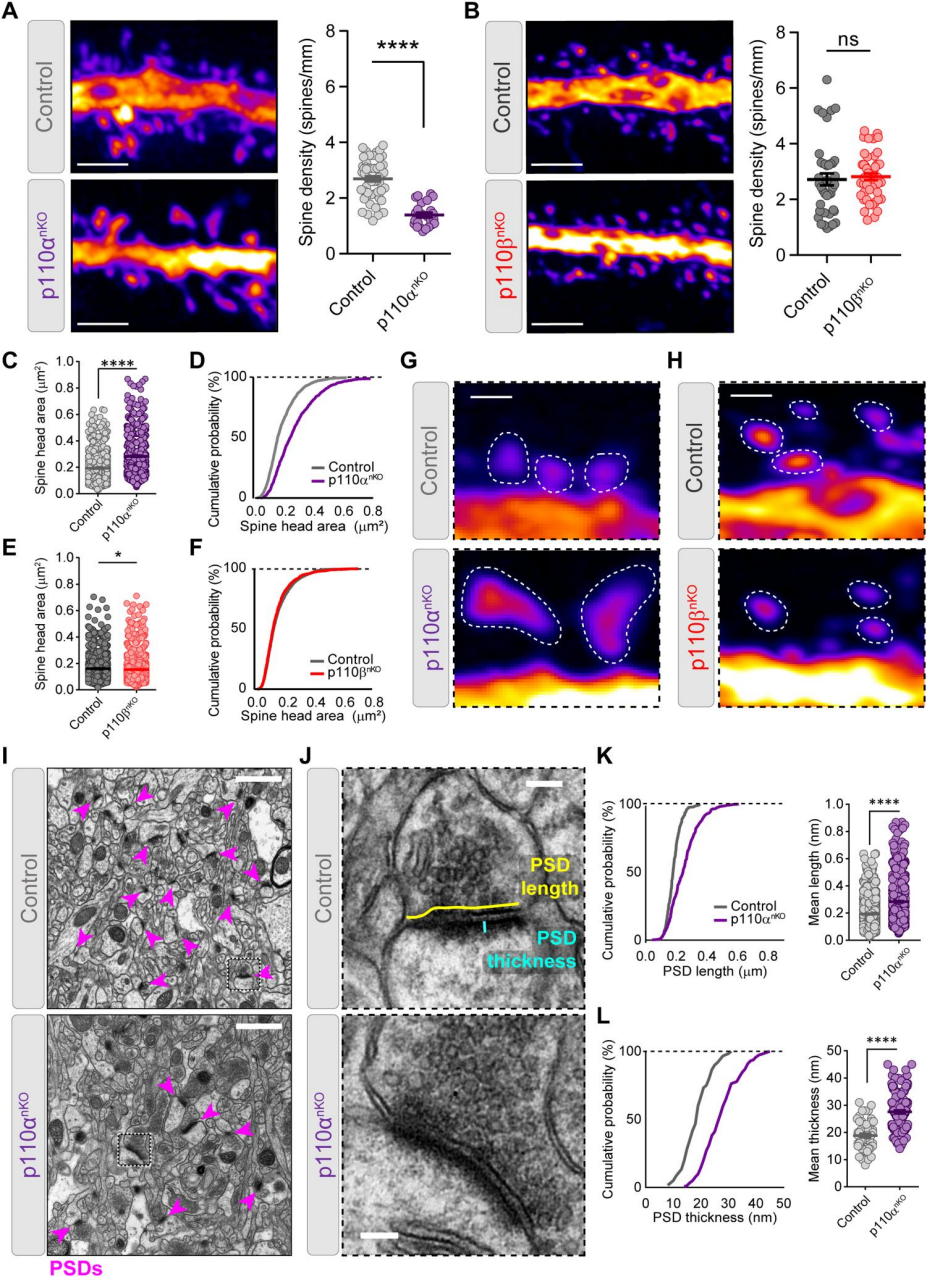
p110α is required to maintain synaptic density and postsynaptic morphology in CA1 neurons. (**A** and **B**) Quantification of spine density from CA1 neuron dendrites from p110α^nKO^ (A) and p110β^nKO^ (B) and their respective saline control mice. Representative confocal images of GFP dendrites are shown in the left side of each graph. Scale bars, 2 μm. Statistical differences between conditions were assessed by Mann-Whitney test: (A) ****P < 0.0001 versus control and (B) *P* = 0.314 versus control. p110α*^flox/flox^*: vehicle, *n* = 54; p110α^nKO^, *n* = 24 dendrites. p110β*^flox/flox^*: vehicle, *n* = 40; p110β^nKO^, *n* = 47 dendrites, from five mice per condition. (**C** to **F**) Quantification of mean spine head area and cumulative distributions from neurons of p110α^nKO^ (C and D) and p110β^nKO^ (E and F) mice. Data were analyzed by Mann-Whitney test: (C) ****P < 0.0001 versus control and (E) *P < 0.05 versus control. p110α*^flox/flox^*: vehicle, *n* = 1422; p110α^nKO^, *n* = 792 spines. p110β*^flox/flox^*: vehicle, *n* = 1503; p110β^nKO^, *n* = 1457 spines, from three mice per condition. (**G** and **H**) Representative spines for the quantifications shown in (C) to (F). Scale bars, 0.5 μm. (**I** and **J**) Representative electron micrograph examples from CA1 stratum radiatum of control and p110α^nKO^ mice. Higher-magnification pictures from representative synapses are shown in (J), with drawings indicating calculation of postsynaptic density (PSD) length and thickness. Arrowheads in pink indicate examples of PSDs. Scale bars, 1 μm (I) and 0.2 μm (J). (**K** and **L**) Cumulative frequency distributions (left) and scatter dot plots (right) for PSD length (K) and PSD thickness (L) in control and p110α^nKO^ synapses. Data were compared by Mann-Whitney test (****P < 0.0001 versus control). Number of synapses on each experiment (control and p110α^nKO^, three mice per condition): *n* = 250 and 336 (PSD length) and *n* = 60 and 144 (PSD thickness). Data are shown as means + SEM.

We then analyzed spine density and morphology from GFP-expressing neurons in p110α^nKO^ and p110β^nKO^ mice. p110α deletion also promoted a marked reduction in spine density, which remained unaltered in the absence of p110β (Fig. 3, A and B). Remaining spines from p110α^nKO^ mice were wider and had larger head area as compared to control mice (Fig. 3, C, D, and G; and fig. S6, A and B), without changes in spine length (fig. S6C). In contrast, the absence of p110β had a very minor (although statistically significant) reduction of the spine head area (Fig. 3, E, F, and H) without changes in spine width or length (fig. S6, D to F). Next, we evaluated synaptic structure in more detail in the CA1 region of the hippocampus (stratum radiatum) by performing electron microscopy imaging of synapses in p110α^nKO^ and p110β^nKO^. As shown in Fig. 3 (I to L), compared to controls, spines from p110α^nKO^ neurons had longer and thicker postsynaptic densities. In contrast, the length and thickness of postsynaptic densities from p110β^nKO^ mice were not altered, as compared to controls (fig. S7).

Together, these results evidence that absence of p110α leads to a marked remodeling of neuronal and synaptic morphology, with a major retraction of dendritic arborization and loss of spines, which is accompanied by an enlargement of remaining postsynaptic structures. These changes are much more modest in p110β^nKO^ neurons, indicating that PI3K-dependent regulation of neuronal morphology and postsynaptic structure is mainly mediated by the p110α isoform.

### Absence of p110α and p110β causes an accumulation of vesicles at the presynaptic terminals

**Fig. 4.**
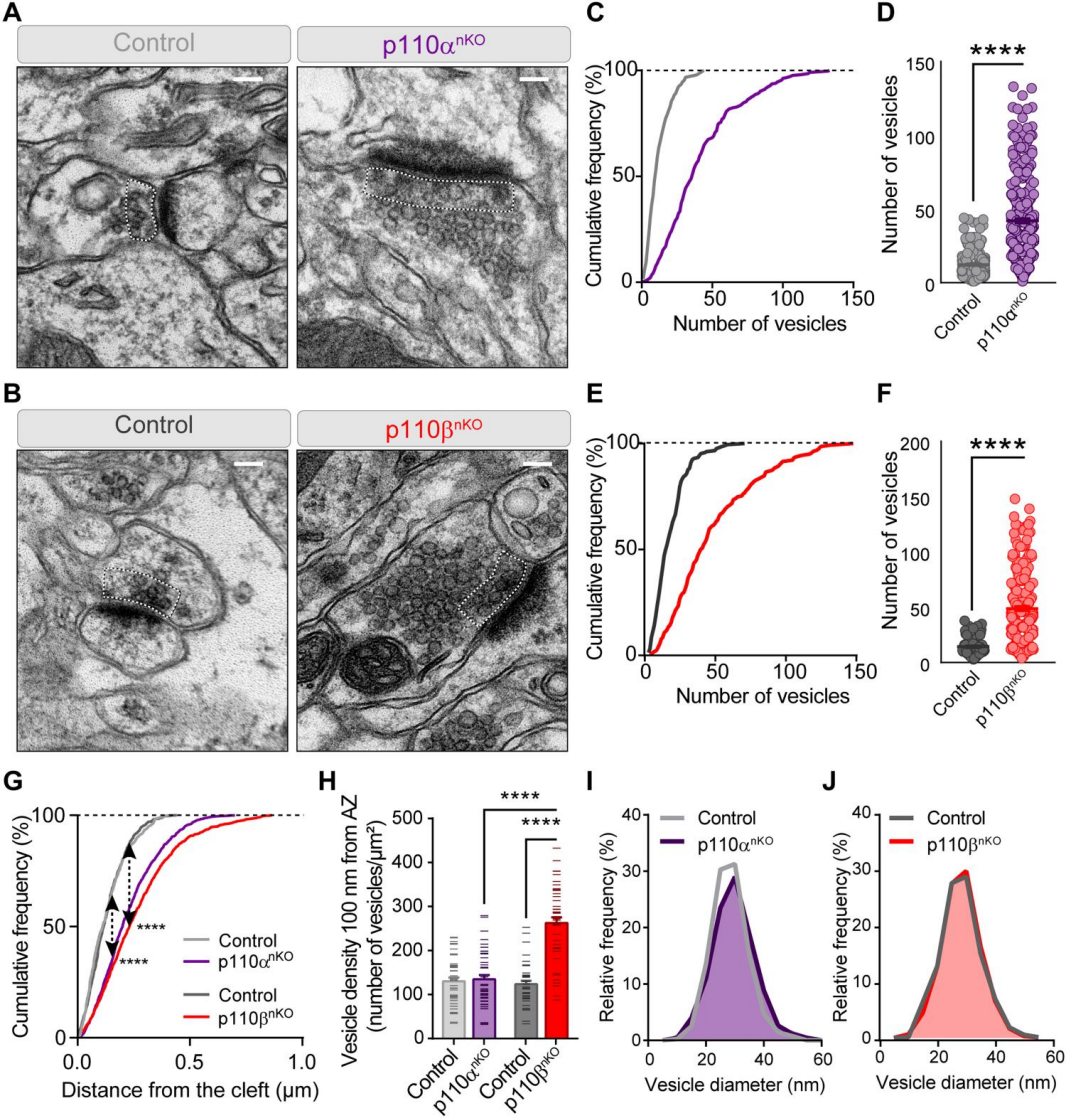
Lack of p110α or p110β results in an accumulation of presynaptic vesicles. (**A** and **B**) Representative electron micrograph examples from CA1 stratum radiatum of p110α^nKO^ (A) and p110β^nKO^ (B) and their respective controls. Dotted lines depict the area within 100 nm of the synaptic cleft that was used for quantification in (H). Scale bars, 0.1 μm. (**C** to **F**) Cumulative distributions and scatter plots for the number of vesicles per presynaptic terminal in p110α^nKO^ (C and D) and p110β^nKO^ (E and F). ****P < 0.0001 versus control mice from Mann-Whitney test. Number of synapses: control, *n* = 249; p110α^nKO^, *n* = 347; control, *n* = 213; p110β^nKO^, *n* = 283. (**G**) Cumulative frequency distribution of the vesicle distance to the synaptic cleft. ****P < 0.0001 from Kolmogorov-Smirnov test (arrows depict Kolmogorov-Smirnov statistic). Number of synaptic vesicles: 466 (control p110α, from 40 presynaptic terminals), 1302 (p110α^nKO^, from 30 presynaptic terminals), 690 (control p110β, from 40 presynaptic terminals), and 1080 (p110β^nKO^, from 20 presynaptic terminals). (**H**) Bar plots for the vesicle density within 100 nm from the synaptic cleft. ****P < 0.0001 versus control from Mann-Whitney test. Number of synapses on each experiment (two mice per condition): *n* = 45 and 42 (control p110α and p110α^nKO^) and *n* = 44 and 37 (control p110β and p110β^nKO^). (**I** and **J**) Frequency histograms of synaptic vesicle size from p110α (I) (purple area) and p110β (J) (red area) knockout neurons and their respective controls (gray lines). Vesicle size was measured from electron microscopy pictures where the active zone membrane was clearly visible. p110α*^flox/flox^*: vehicle, *n* = 99; p110α^nKO^: *n* = 134 synapses. p110β*^flox/flox^*: vehicle *n* = 50; p110β^nKO^: *n* = 109 synapses. Three mice per condition for the AAV infections and one or two mice for the vehicle.

The electron microscopy analysis presented above also allowed us to investigate potential alterations at presynaptic terminals in neurons lacking p110α or p110β. As shown in Fig. 4 (A to F), both p110α^nKO^ and p110β^nKO^ neurons showed a marked increase (about threefold) in the number of synaptic vesicles per synapse, as compared to neurons from control mice. These vesicles spread throughout larger distances from the synaptic cleft, as shown in the cumulative distributions in Fig. 4G. To get further insight into the potential functional relevance of this vesicle accumulation, we then quantified synaptic vesicle density in close proximity (100 nm) to the synaptic membrane as a morphological approximation to the pool of readily releasable vesicles (*41*). In this case, we observed a statistically significant increase (about twofold) in vesicle density for p110β^nKO^ neurons, while there were no differences in p110α^nKO^ neurons, as compared to their controls (Fig. 4H). The difference in synaptic vesicle density in this region was also statistically different between p110α^nKO^ and p110β^nKO^ conditions. To note, these measurements are expressed as vesicle density, therefore normalizing for differences in the length of the synaptic membrane. On the other hand, the diameter of the presynaptic vesicles was not altered in p110α^nKO^ or p110β^nKO^ neurons (Fig. 4, I to J). Overall, these analyses support a more prominent role at the presynaptic terminal for p110β, as compared to p110α.

### p110α and p110β control basal synaptic transmission in opposite directions

**Fig. 5.**
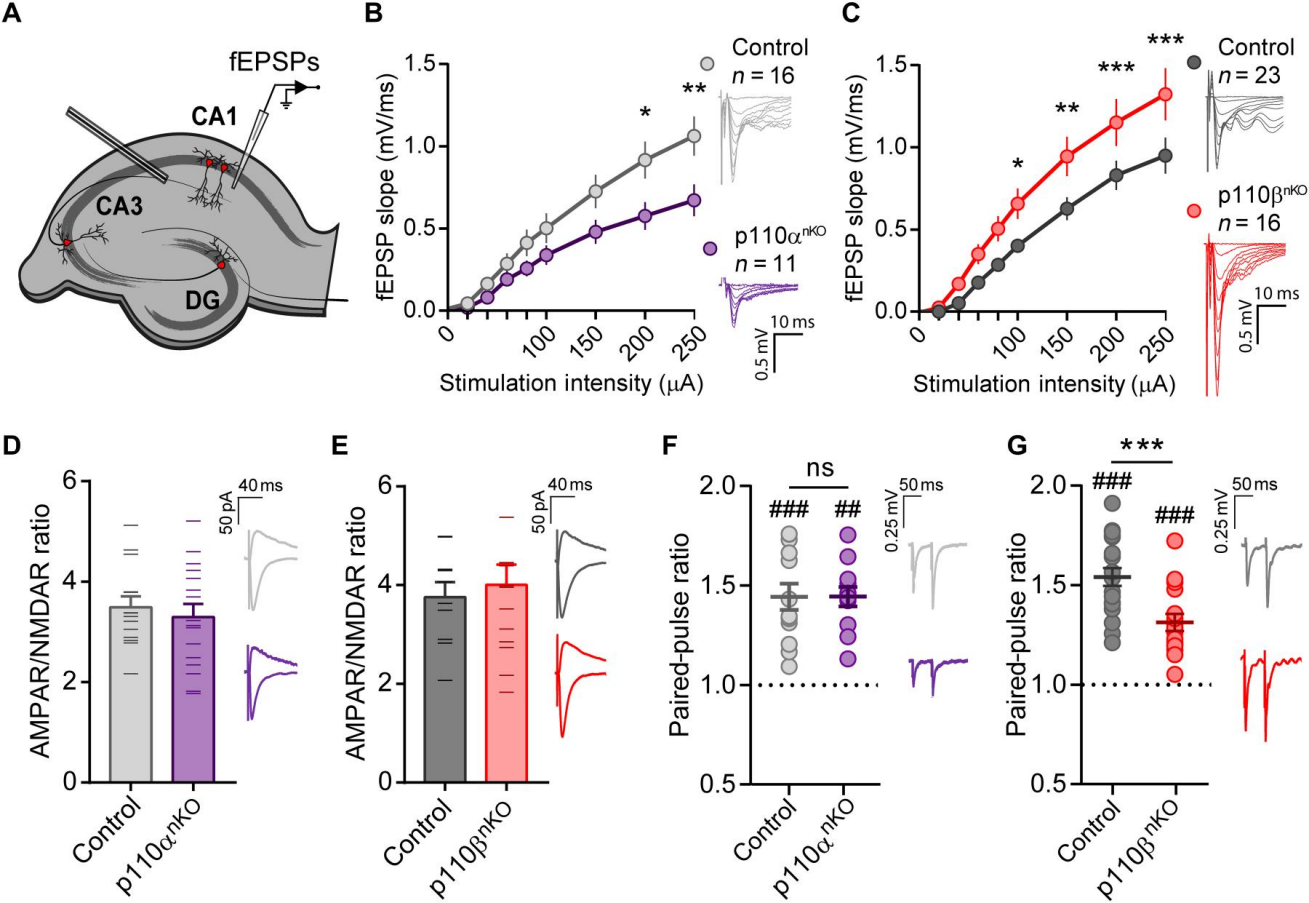
Neuronal p110α and p110β isoforms have different effects on basal transmission. (**A**) Experimental configuration for extracellular field recordings in acute slices. DG, dentate gyrus. (**B** and **C**) Input-output curves of the extracellular fEPSPs from p110α^nKO^ (B) and p110β^nKO^ (C) slices evoked at different stimulation intensities. p110α^nKO^: *n* = 16 slices from five mice; two-way repeated-measures ANOVA, *F*(8,200) = 4.177, *P* = 0.0001. p110β^nKO^: *n* = 18 to 16 slices from five mice; two-way repeated-measures ANOVA, *F*(8,256) = 5.97, *P* < 0.0001. Bonferroni’s posttest (*P < 0.05; **P < 0.01; ***P < 0.005). Representative traces from one experiment for each condition are depicted on the right. Scale bar, 0.5 mV, 10 ms. (**D** and **E**) Bar graph of the ratio of AMPAR- to NMDAR-mediated excitatory postsynaptic currents (EPSCs) in CA1 pyramidal neurons from p110α^nKO^ and p110βn^KO^ mice recorded on whole-cell voltage clamp configuration. AMPAR responses were measured at −60 mV and NMDAR responses at +40 mV, 60 ms after stimulation of the Schaffer collaterals. p110α*^flox/flox^*: control, *n* = 14; p110α^nKO^, *n* = 16 cells. p110β*^flox/flox^*: control, *n* = 10; p110β^nKO^, *n* = 14 cells from three mice per condition. Representative traces are shown on the right of each graph. Scale bars, 50 pA, 40 ms. No statistically significant differences were observed by two-tailed nonparametric Mann-Whitney test. (**F** and **G**) Average values of PPRs from fEPSP recordings from p110α^nKO^ (F) and p110β^nKO^ (G) slices with 50-ms interstimulus interval. Individual values for each condition are displayed as a dot plot. p110α*^flox/flox^*: vehicle, *n* = 13; p110α^nKO^, *n* = 12 slices. p110β*^flox/flox^*: vehicle, *n* = 19; p110β^nKO^, *n* = 16 slices from more than four mice per condition. Representative traces for each condition are shown on the right of each panel. Statistical differences between conditions were assessed with Mann-Whitney test (***P < 0.001). Wilcoxon matched-pairs signed-rank test was used to assess significant paired-pulse facilitation (##*P* < 0.01 and ###*P* < 0.005).

Our morphological data indicate that p110α and p110β are differentially required for maintaining proper neuronal morphology at both postsynaptic and presynaptic level. To evaluate the functional impact of these morphological observations, we first evaluated basal synaptic transmission by extracellular recordings of Schaffer collateral–CA1 synapses from acute hippocampal slices of p110α^nKO^ and p110β^nKO^ mice (see Fig. 5A for experimental configuration). Field excitatory postsynaptic potentials (fEPSPs) were measured in response to increasing stimulus intensities to generate input/output curves. We found that the genetic deletion of p110α and p110β caused opposite effects on basal synaptic transmission. Thus, slices from p110α^nKO^ mice displayed a decrease in synaptic responses (Fig. 5B), whereas the absence of p110β produced an enhancement of synaptic transmission (Fig. 5C). None of these manipulations altered the excitability of the Schaffer collateral axons, as determined by presynaptic fiber volley amplitude (fig. S8, A and B). We did not find any differences in the ratio of AMPAR to NMDAR responses in whole-cell voltage-clamp recordings of CA1 neurons from p110α and p110β mice (Fig. 5, D and E), suggesting that AMPAR- and NMDAR-mediated synaptic transmissions were altered to the same extent in p110α^nKO^ and p110β^nKO^ CA1 neurons. Next, as a measurement of presynaptic function, we evaluated the paired-pulse ratio (PPR) of fEPSPs in hippocampal CA1 pyramidal neurons in response to two consecutive stimulations (50 ms apart). When p110α was removed, PPR remained similar to control slices (Fig. 5F), while PPR was significantly reduced in p110β^nKO^ slices (Fig. 5G). These results suggest that the absence of p110β produces an increased probability of glutamate release. These results are in good agreement with the enhanced synaptic transmission in p110β-lacking slices (Fig. 5C) and may also fit with the accumulation of neurotransmitter vesicles in close proximity to the presynaptic membrane, as observed in our morphological analysis (Fig. 4H). Incidentally, the decreased basal transmission in p110α-lacking slices is also consistent with the marked reduction in spine density in these animals (Fig. 3A). Overall, this initial electrophysiological characterization reinforces the notion of a prevalent role of the p110β isoform in presynaptic mechanisms, while the p110α isoform would exert its actions on neuronal architecture and postsynaptic structure.

### p110α and p110β control different forms of synaptic plasticity

**Fig. 6.**
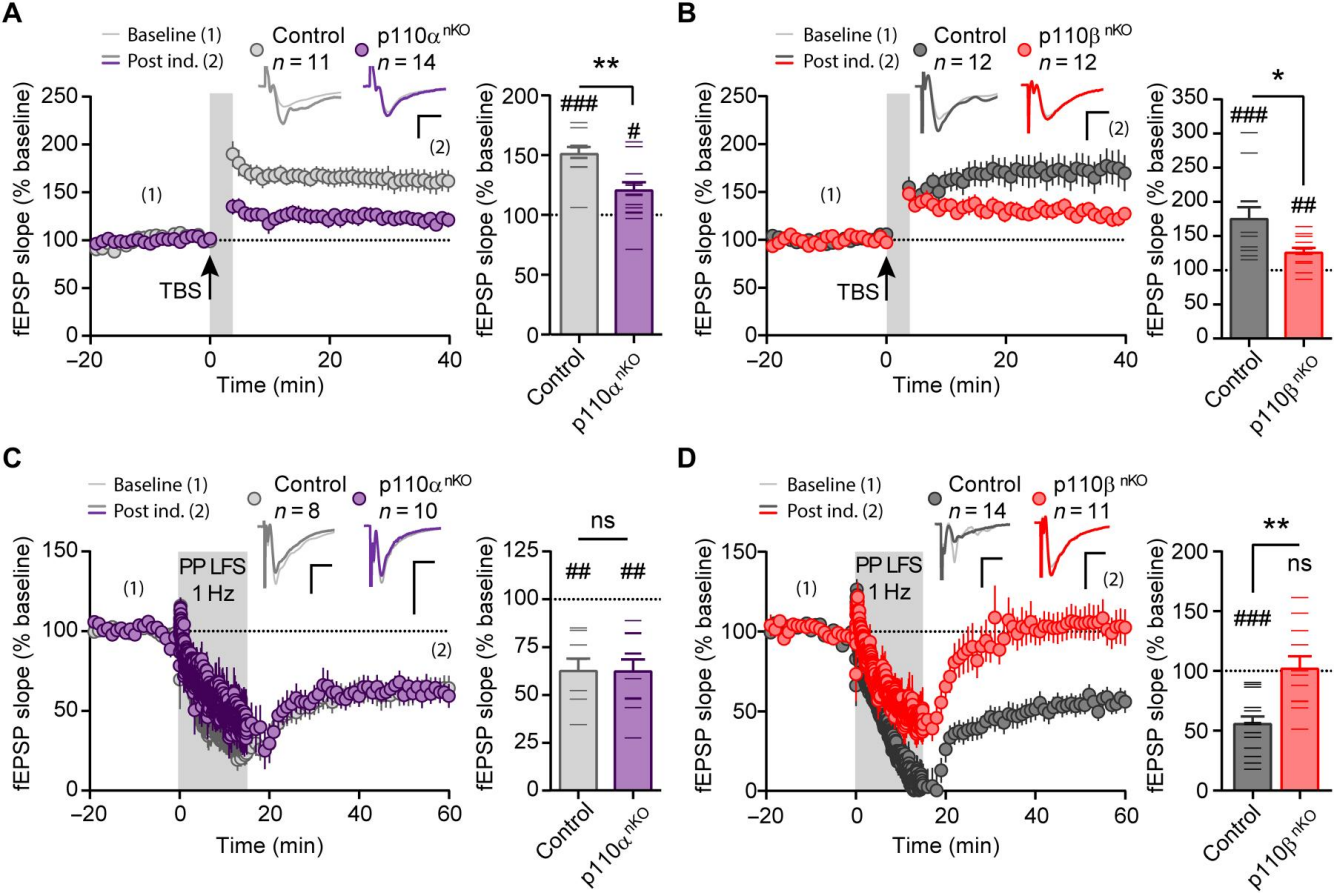
Neuronal p110α and p110β isoforms are differentially required for synaptic plasticity. (**A** and **B**) Time course of NMDAR-dependent LTP induced by TBS in p110α^nKO^ (A) and p110β^nKO^ (B) slices and their respective control mice. Wilcoxon statistical test was used to evaluate LTP expression with respect to baseline (#*P* < 0.05; ##*P* < 0.01; ###*P* < 0.005). Right: Bar graphs showing the average change in fEPSP slope from the last 5 min of the recording. Statistical differences between conditions were assessed with Mann-Whitney test (*P < 0.05; **P < 0.01). p110α*^flox/flox^*: vehicle, *n* = 13; p110α^nKO^, *n* = 12 slices. p110β*^flox/flox^*: vehicle, *n* = 19; p110β^nKO^, *n* = 16 slices from more than four mice per condition. Representative traces are shown above the graphs. (**C** and **D**) Time course of mGluR-dependent LTD induced by paired-pulse low-frequency stimulation (PP LFS) in the presence of APV in p110α^nKO^ (C) and p110β^nKO^ slices (D) and their respective control mice. Right: Bar graphs showing the average fEPSP slope from the last 5 min of recording. Wilcoxon statistical test was used to evaluate LTD expression with respect to baseline (##*P* < 0.01; ###*P *< 0.005). Statistical differences between conditions were assessed with Mann-Whitney test (**P < 0.01). p110α*^flox/flox^*: vehicle, *n* = 13; p110α^nKO^, *n* = 12 slices. p110β*^flox/flox^*: vehicle, *n* = 19; p110β^nKO^, *n* = 16 slices from more than four mice per condition. Representative traces are shown above the graphs. For all panels, data are presented as means ± SEM.

We next examined the contribution of p110α and p110β to two forms of synaptic plasticity that have been shown to require PI3K activity, namely, NMDAR-dependent LTP and mGluR-dependent LTD (*12*–*18*, *42*). LTP was induced by theta-burst stimulation (TBS) and mGluR-LTD by paired-pulse low-frequency stimulation in the presence of d,l-2-amino-5-phosphonovaleric acid (APV; NMDAR antagonist), in acute hippocampal slices from p110α^nKO^ and p110β^nKO^ mice. We found that the absence of both p110α and p110β significantly impaired LTP expression when compared to their respective control mice (Fig. 6, A and B). In contrast, mGluR-LTD was selectively abolished in slices from p110β^nKO^ mice (Fig. 6, C and D), indicating the specific requirement of the p110β isoform for mGluR-LTD. In addition, neither p110α nor p110β were required for NMDAR-dependent LTD (fig. S8, C and D), in agreement with previous studies (*20*).

**Fig. 7.**
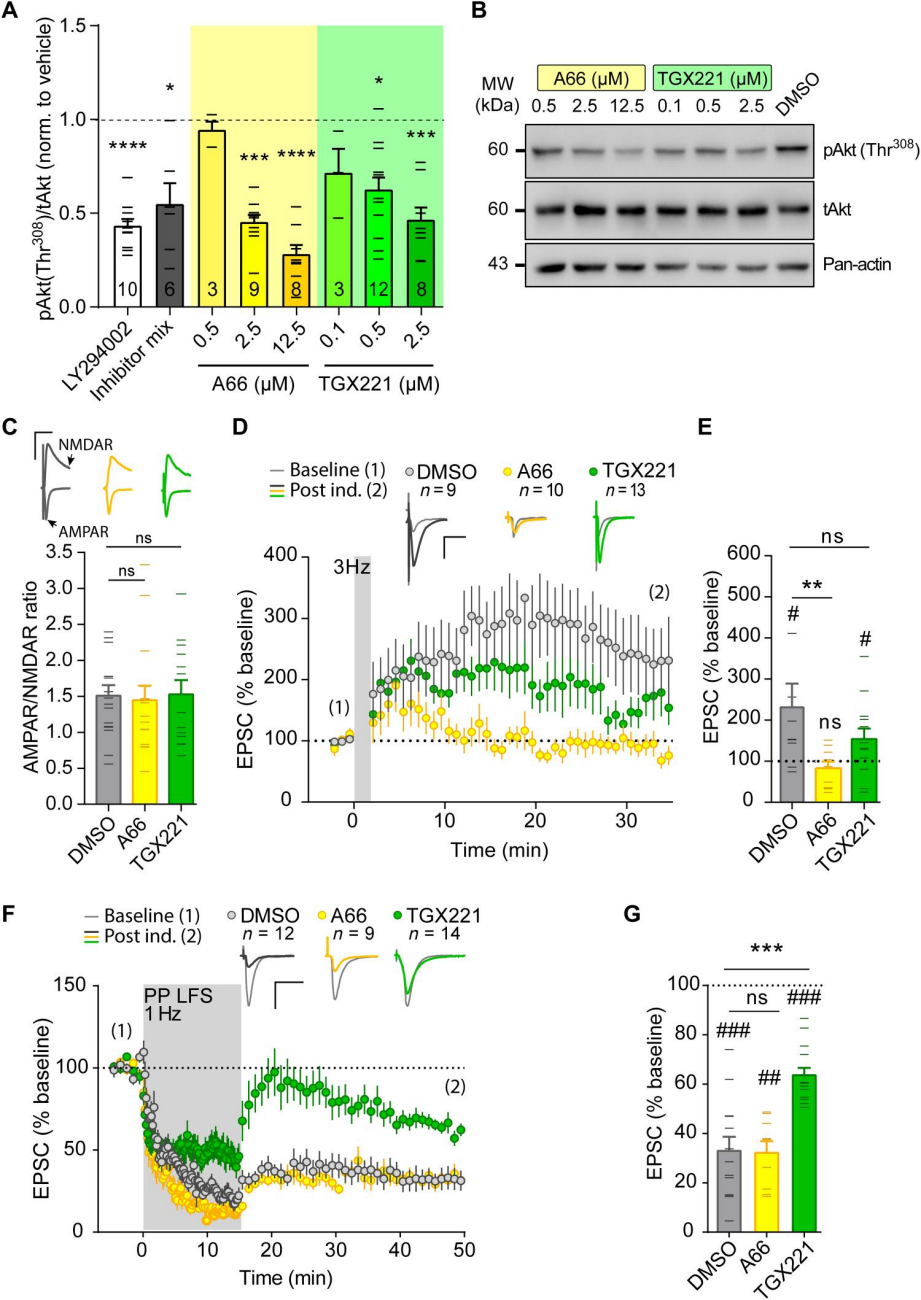
Effect of pharmacological inhibition of p110α and p110β on basal transmission and synaptic plasticity. (**A**) Quantification of the phospho/total ratio for Akt(Thr^308^) [pAkt(Thr^308^)/tAkt] from Western blots of whole extracts from organotypic slice cultures treated overnight with different concentrations of the p110α-selective inhibitor (A66) or the p110β-selective inhibitor (TGX221), the nonselective PI3K inhibitor (10 μM LY294002), or an inhibitor mix (0.5 μM A66, 0.1 μM TGX221, and 0.02 μM CAL101-p110δ–selective inhibitor). Statistical differences were assessed by Kruskal-Wallis nonparametric test and Dunn’s multiple comparison test (*P < 0.05, ***P < 0.001, and ****P < 0.0001). (**B**) Representative images of Western blots. MW, molecular weight. (**C**) AMPAR/NMDAR ratios from slices treated as indicated. *n* = 15 [dimethyl sulfoxide (DMSO)], 14 (A66), 15 (TGX221) cells from *n* > 4 rats per condition. (**D** and **F**) Time course of normalized AMPAR-mediated EPSCs from baseline and after induction of NMDAR-dependent LTP (D) or mGluR-LTD (F). Representative traces before induction (1; thin lines) and for the last 5 min of the time course (2; thick lines) are depicted above the time course. Vertical gray shading indicates the induction protocol. (**E** and **G**) Summary bar plot of average EPSC amplitude for each cell from the last 5 min of recording. Wilcoxon matched-pairs signed-rank test (#) was used to assess statistically significant potentiation. Mann-Whitney test (*) was used to evaluate significant differences between conditions. **P < 0.01, ***P < 0.001, #*P*< 0.05, ##*P* < 0.01, and ###*P* < 0.001. For all panels, data are presented as means ± SEM.

To bypass potential effects of the semichronic genetic deletion of p110α or p110β, we used the isoform-specific inhibitors A66 and TGX221 (*43*, *44*) to pharmacologically suppress the catalytic activity from the p110α and p110β isoforms, respectively, in a more acute manner (Fig. 7) (to note, bath application of PI3K inhibitors is not restricted to neurons, as it is the case with the p110α^nKO^ or p110β^nKO^ mice). We chose the concentrations of 2.5 μM for A66 and 0.5 μM for TGX221 based on their effect on PI3K pathway activation, as reported by phosphorylation of Akt (pAkt) at Thr^308^ (Fig. 7, A and B). Similar to our previous results with acute slices from p110α^nKO^ and p110β^nKO^ mice, we did not find any differences in AMPAR/NMDAR ratios from A66-, TGX221-, and vehicle-treated slices (Fig. 7C). NMDAR-dependent LTP was evaluated under whole-cell voltage-clamp configuration by pairing presynaptic stimulation of Schaffer collaterals (3 Hz) with postsynaptic depolarization (0 mV) of CA1 neurons (*45*–*47*). Under this configuration, LTP was abolished in A66-treated slices. On the other hand, TGX221 appeared to reduce the extent of synaptic potentiation, although the effect was not statistically significant (Fig. 7, D and E). mGluR LTD was also carried out under whole-cell voltage-clamp configuration with low-frequency paired pulses of Schaffer collaterals. In this case, the p110β inhibitor (TGX221) completely abolished mGluR-LTD expression, whereas the p110α inhibitor (A66) did not have any effect (Fig. 7, F and G). Therefore, these results with pharmacological inhibition of p110α and p110β isoforms essentially reproduce those obtained with p110α^nKO^ and p110β^nKO^ mice, supporting the interpretation of a selective requirement of p110β for mGluR LTD and pointing to a joint contribution of p110α and p110β to LTP (perhaps with a preferential role of p110α).

### Presynaptic or postsynaptic loci of action for p110α and p110β during synaptic plasticity

**Fig. 8.**
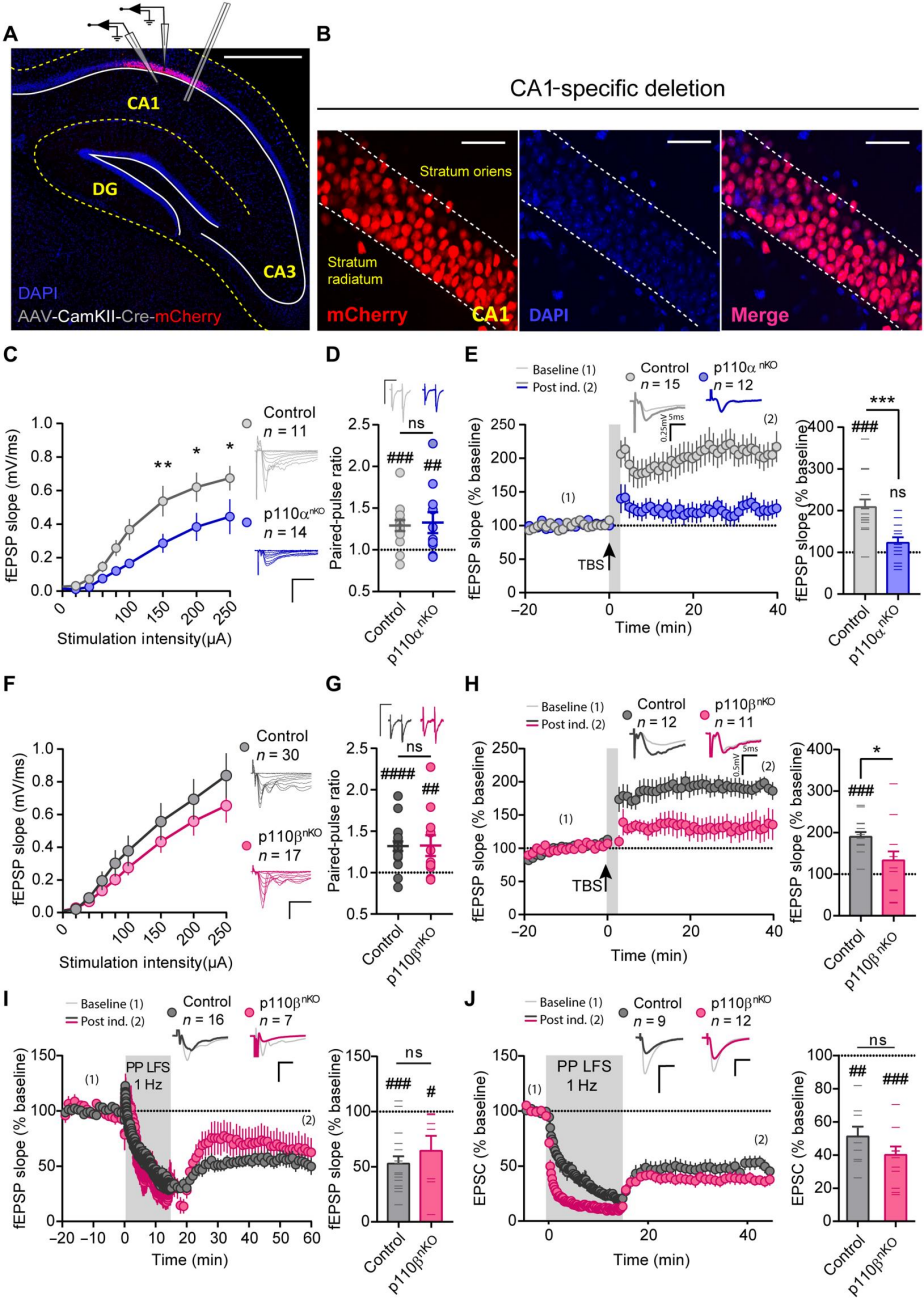
Synaptic function and plasticity after postsynaptic removal of p110α or p110β. (**A** and **B**) Representative images of CA1-infected hippocampus, as identified from mCherry fluorescence, and experimental configuration for extracellular fEPSP and whole-cell EPSC recordings. Scale bars, 500 μm (A) and 50 μm (B). (**C** and **F**) Input-output curves of fEPSP slopes versus stimulation intensity. (C) Control, *n* = 11; p110α^nKO^, *n* = 14 slices from four mice; two-way repeated-measures ANOVA, *F*(8,124) = 2.845, *P* = 0.005. (F) Control, *n* = 30; p110β^nKO^, *n* = 17 slices from more than five mice; two-way repeated-measures ANOVA, ns. Bonferroni’s posttest (*P < 0.05 and **P < 0.01). Representative traces are depicted on the right of the plots. (**D** and **G**) Quantification of PPRs of fEPSPs. Representative traces are shown above the plots. (D) Control, *n* = 15; p110α^nKO^, *n* = 11. (G) Control, *n* = 19; p110β^nKO^, *n* = 11 slices from more than five mice. (**E**, **H**, and **I**) Time course of normalized fEPSP responses from baseline and after induction of NMDAR-dependent LTP (E and H) or mGluR-dependent LTD (I), on p110α^nKO^ (E) or p110β^nKO^ (H and I) slices. Representative traces before induction (1; thin lines) and from the last 5 min of the time course (2; thick lines) are depicted above the time course. Right: Summary bar plot of fEPSP slope from the last 5 min of recording. (**J**) Similar to (I) but with whole-cell voltage-clamp recordings of EPSCs from individual CA1 neurons expressing Cre-mCherry. For all panels, statistical differences between conditions were assessed with Mann-Whitney test (*). Wilcoxon matched-pairs signed-rank test (#) was used to assess statistically significant paired-pulse facilitation (D and G), potentiation (E and H), and depression (I and J). *P < 0.05, ***P < 0.001, #*P* < 0.05, ##*P* < 0.01, ###*P* < 0.005; ####*P* < 0.001. *n*: number of slices (C to I) or number of individual cells (J). Bars represent means ± SEM.

The combination of our proteomic and morphological data, together with the specific effect of p110β in paired-pulse facilitation, suggests that p110α and p110β could be exerting their synaptic functions differentially at the pre- or postsynaptic compartments. To further explore this possibility, we restricted the AAV-CaMKII-Cre injections on p110α and p110β floxed mice to the CA1 hippocampal region (Fig. 8, A and B). This strategy allowed us to specifically remove p110α or p110β from postsynaptic CA1 neurons and explore whether specific changes in basal transmission and synaptic plasticity are still present. Deletion of p110α in CA1 postsynaptic neurons significantly reduced basal transmission (Fig. 8C), blocked NMDAR-dependent LTP (Fig. 8E), and did not alter PPR (Fig. 8D) or fiber volley amplitude (fig. S8E), similar to the results obtained with whole hippocampal infection (Figs. 5 and 6). These results strongly suggest that the synaptic locus of action of p110α is confined within the postsynaptic compartment.

On the other hand, p110β-specific deletion from postsynaptic CA1 neurons no longer altered basal synaptic transmission (Fig. 8F) or PPR (Fig. 8G), in contrast with the results obtained with the full p110β^nKO^ (Fig. 5, C and G). These results suggest that these parameters are controlled by p110β at the presynaptic compartment. To note, fiber volley amplitude was not affected by removal of p110β from postsynaptic CA1 neurons (fig. S8F), similar to the result obtained with the full p110β^nKO^. LTP was still impaired when p110β was removed specifically from CA1 neurons (Fig. 8H), suggesting that p110β is required at the postsynaptic compartment for its contribution to LTP. mGluR-LTD was completely preserved in postsynaptic p110β knockout neurons, as compared to control mice, both from extracellular fEPSP recordings (Fig. 8I) and from whole-cell recordings from individual CA1 knockout (infected) neurons (Fig. 8J). Together, these results point to a dual role of p110β: acting at the presynaptic terminal to control neurotransmitter release and to mediate mGluR-LTD while contributing to NMDAR-dependent LTP at the postsynaptic compartment.

### Neuronal deletion of p110α or p110β in the adult hippocampus causes specific behavioral abnormalities

Given the specific contribution of p110α and p110β isoforms to synaptic plasticity and neuronal architecture, we lastly evaluated whether deletion of neuronal p110α and p110β has cognitive consequences in vivo. To this end, 4 weeks after hippocampal infection with AAV-CaMKII-mCherry-Cre, p110α^nKO^, p110β^nKO^ mice, and their respective controls were tested for different behaviors, ranging from anxiety to memory and social behavior.

**Fig. 9.**
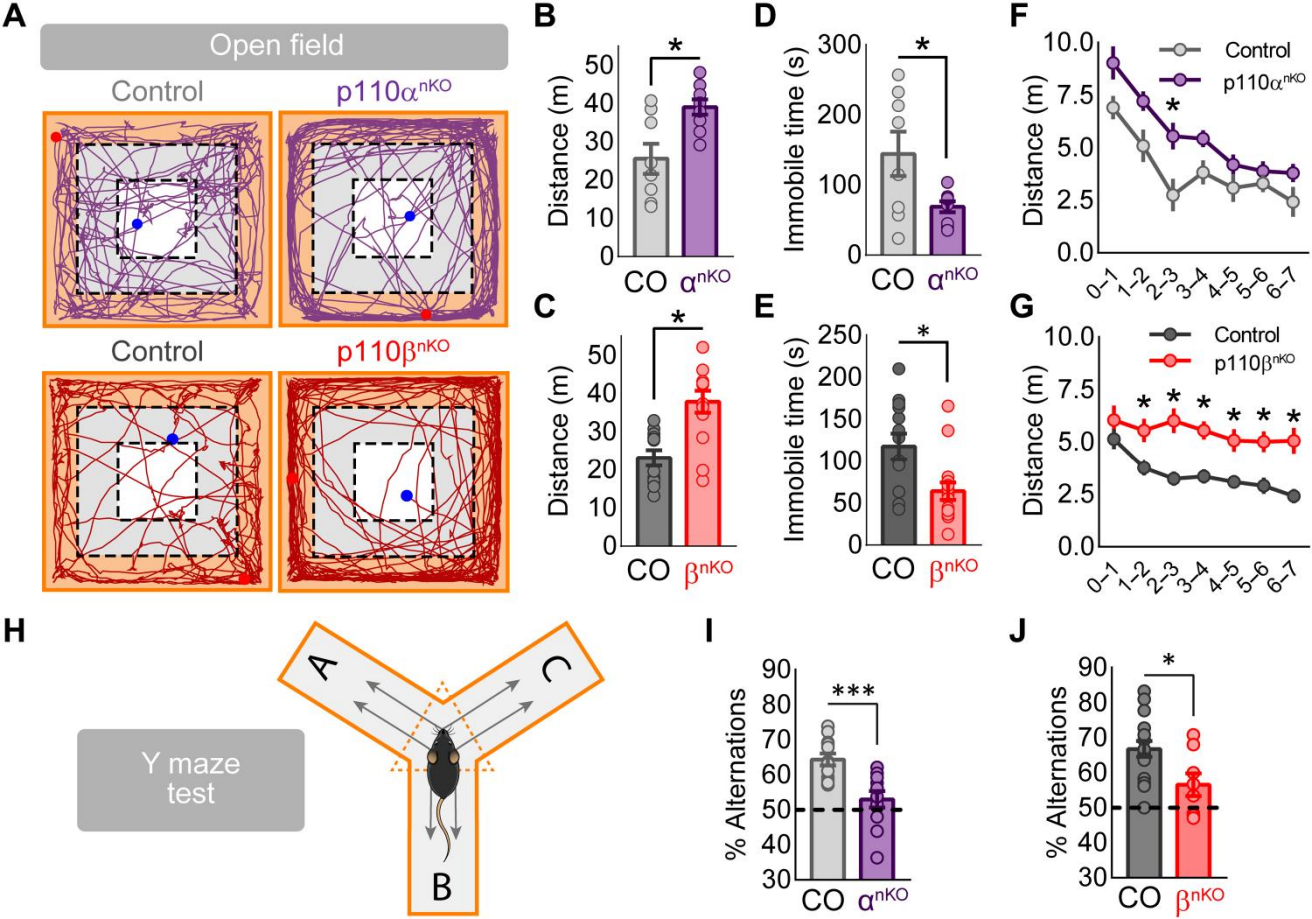
Open field and Y-maze tests on p110α^nKO^ and p110β^nKO^ animals. (**A** to **G**) Mice were tested in the open field for exploring locomotor behavior. Mice were placed individually in the center of an open field arena (40 cm by 40 cm by 40 cm) and allowed to habituate and freely explore the area for 7 min. (A) Representative paths of ambulation from p110α^nKO^ (top), p110β^nKO^ (bottom), and their respective controls for the entire duration of the test. Periphery, inner, and center zones are also shown in orange, gray, and white, respectively. (B and C) Quantification of the total traveled distance for the entire duration of the test for p110α (B) and p110β (C) floxed mice. (D and E) Quantification of the mean time that mice spend immobile in the arena for p110α (D) and p110β (E) floxed mice. (F and G) Quantification of the mean traveled distance in time segments of 1 min for p110α (F) and p110β (G) floxed mice. Mann-Whitney test was used to assess statistical differences between p110α^nKO^ or p110β^nKO^ and controls (*P < 0.05). p110α*^flox/flox^*: vehicle, *n* = 8; p110α^nKO^, *n* = 9. p110β*^flox/flox^*: vehicle, *n* = 13; p110β^nKO^, *n* = 15. (**H**) Experimental scheme of the spontaneous alternation task that is performed on a Y-shaped maze. Mice are placed on the center region of the maze; the arm entrance order is recorded, and then the spontaneous alternation percentage is calculated. (**I** and **J**) Spontaneous alternation percentages from p110α^nKO^ (I) and p110β^nKO^ (J) and their respective controls. Statistical analysis between conditions was performed with Mann-Whitney test (***P < 0.001 and *P < 0.05). p110α*^flox/flox^*: control, *n* = 12; p110α^nKO^, *n* = 12. p110β*^flox/flox^*: control, *n* = 16; p110β^nKO^, *n* = 8.

Spontaneous locomotor activity was evaluated in the open field test (see representative mouse trajectories in Fig. 9A). Both p110α^nKO^ and p110β^nKO^ mice showed higher locomotor activity than control mice, which was reflected in longer distances traveled (Fig. 9, B and C), faster movement (fig. S9, A and B), and reduced immobility times (Fig. 9, D and E), as compared to control mice. The hyperlocomotion phenotype was qualitatively different between p110α^nKO^ and p110β^nKO^ mice. Thus, p110α^nKO^ mice were hyperactive but showed habituation to the novel environment (reduction in locomotor activity over time) in the first few minutes (Fig. 9F). In contrast, p110β^nKO^ mice continued being hyperactive for the whole duration of the test, suggesting a defective habituation when p110β was removed (Fig. 9G). The open field arena was also used to investigate basal anxiety levels (see definition of experimental zones in fig. S9C). We observed that the hyperlocomotion of both p110α^nKO^ and p110β^nKO^ mice preferentially occurred at the periphery of the arena, where they traveled significantly longer distances (fig. S9, D and E) and spent longer time (fig. S9, F and G) than control mice. On the other hand, a more detailed analysis revealed an increase in exploratory activity for both p110α^nKO^ and p110β^nKO^ mice in the borders of the arena, specifically rearing and olfactory investigation of the borders (fig. S9, H and I).

The increase in locomotion at the periphery of the open field could suggest an increased anxiety. Therefore, we next tested p110α^nKO^ and p110β^nKO^ mice in the elevated plus maze as a more direct assay to evaluate anxiety. However, using this test, we did not find any significant difference between control and p110α^nKO^ or p110β^nKO^ mice in traveled distance (fig. S10A), time spent (fig. S10B), or number of visits (fig. S10C) to open or closed arms. Therefore, according to this assay and the increase in exploratory activity in the open field, deletion of p110α or p110β isoforms does not appear to produce anxiogenic effects.

We next evaluated working memory in p110α^nKO^ or p110β^nKO^ mice with the alternation test in the Y-maze. In this task, while exploring the maze, control mice typically alternated between the three arms and avoided repetitions of two arms (see schematic cartoon for the test in Fig. 9H). In contrast, both p110α^nKO^ and p110β^nKO^ mice showed a marked reduction in the number of alternations, which may be indicative of impaired working memory or behavioral flexibility (Fig. 9, I and J) (*48*).

**Fig. 10.**
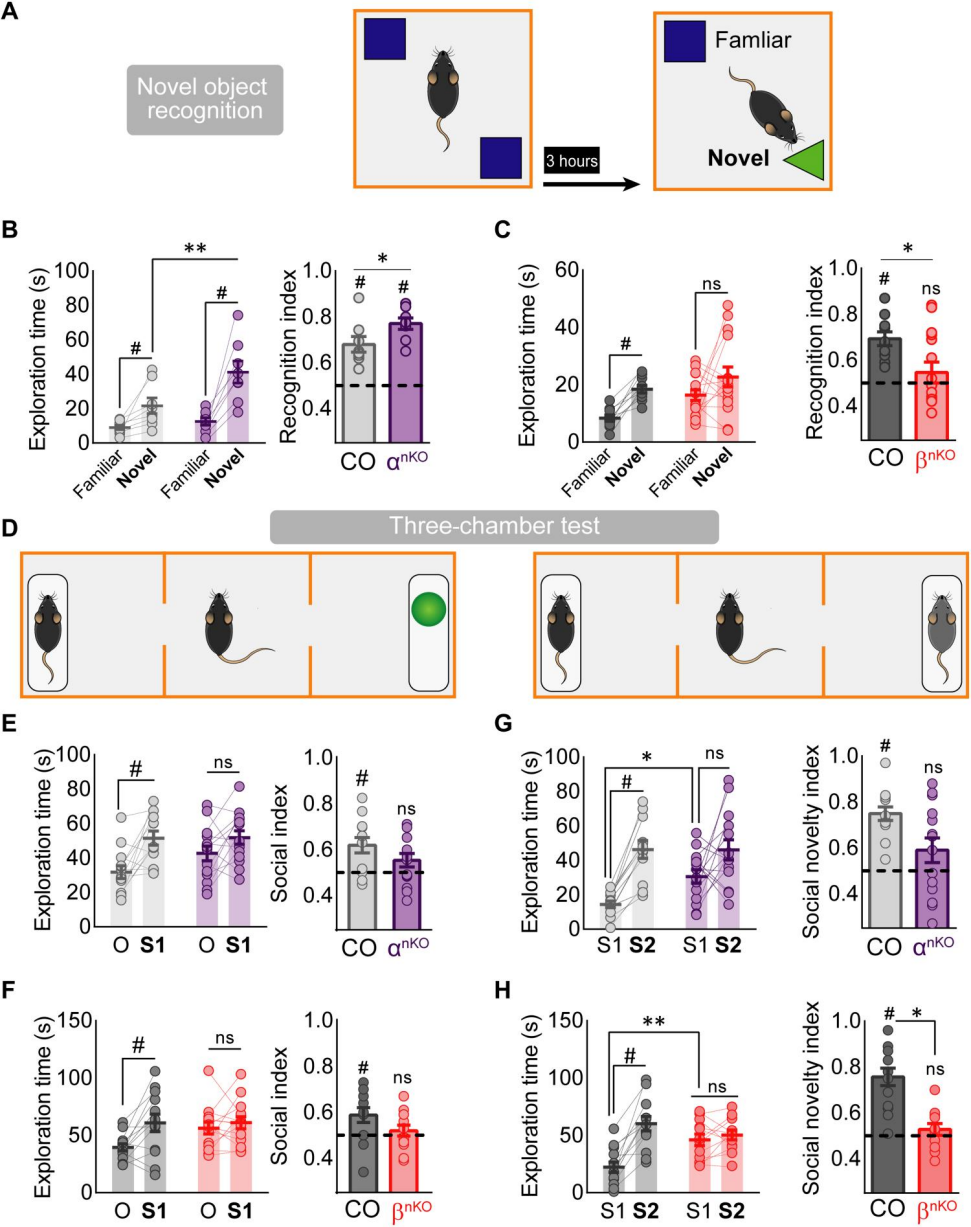
Novel object recognition and sociability tests on p110α^nKO^ and p110β^nKO^ animals. (**A**) Diagram of the NOR test. (**B** and **C**) Left graph: Exploration time of novel and familiar objects for p110α (B) and p110β (C) floxed mice. Statistical significance according to two-way repeated-measures ANOVA [*F*(1,14) = 5.636, *P* = 0.036; Bonferroni’s posttest: **P < 0.01, *P < 0.05]. Recognition index for p110α (B) and p110β (C) floxed mice. Wilcoxon statistical test was used to assay discrimination (#*P* < 0.05), and data between groups was analyzed by two-tailed Mann-Whitney test (*P < 0.05). p110α*^flox/flox^*: vehicle, *n* = 8; p110α^nKO^, *n* = 8. p110β*^flox/flox^*: vehicle, *n* = 9; p110β^nKO^, *n* = 13. (**D**) Schematic representation of the three-chamber social interaction task for the sociability (left) and for the social novelty (right) tests. (**E** and **F**) Exploration time for the subject (S1) versus the object (O), for p110α^nKO^ [(E) left graph; two-way repeated-measures ANOVA, *F*(1,25) = 14.65, *P* = 0.0008] and p110β^nKO^ (F) left graph; two-way repeated-measures ANOVA, *F*(1,25) = 7.836, *P* = 0.01). Bonferroni’s posttest, #*P* < 0.05. Right graphs: Social index (percentage of time exploring the subject over the total exploration time) for p110α^nKO^ (E) and p110β^nKO^ (F) mice. Wilcoxon statistical test was used to assay social preference (#*P* < 0.05). (**G** and **H**) Exploration time of a novel (S2) versus a familiar (S1) mouse for p110α^nKO^ [(G) left graph; two-way repeated-measures ANOVA, *F*(1,24) = 23.87, *P* < 0.0001] and p110β^nKO^ [(H) left graph; two-way repeated-measures ANOVA, *F*(1,23) = 31.11, *P* < 0.0001] mice. Bonferroni’s posttest: *P < 0.05; **P < 0.01. Right graphs: Social novelty index (percentage of time exploring the novel mouse over the total exploration time) for p110α^nKO^ (G) and p110β^nKO^ (H) mice. Wilcoxon statistical test was used to assay social novelty preference (#*P* < 0.05). p110α*^flox/flox^*: vehicle, *n* = 13; p110α^nKO^, *n* = 14. p110β*^flox/flox^*: vehicle, *n* = 13; p110β^nKO^, *n* = 14.

Next, we tested mice for spatial and episodic memory using the novel object location (NOL; fig. S11A) and novel object recognition (NOR; Fig. 10A) tests (NOLT and NORT, respectively). In the NOL task, both p110α^nKO^ and p110β^nKO^ mice displayed normal spatial learning compared to control mice (fig. S11, B and C), as indicated from the preference to explore the object located in a new location. In contrast, in the NORT, we found that while control and p110α^nKO^ mice preferentially explored the novel object (Fig. 10B), p110β^nKO^ mice failed to discriminate the novel versus the familiar object (Fig. 10C). This is reflected in the time exploring the new versus the familiar object and is quantified by the recognition index (Fig. 10, B and C). These results suggest that p110β, but not p110α, is required for object recognition memory. In addition, both p110α^nKO^ and p110β^nKO^ mice displayed normal habituation after object reexposure, as compared to their saline-injected controls (fig. S11, D to F).

Last, we evaluated whether the absence of p110α or p110β alters social behavior. For that purpose, we used the three-chamber sociability and social novelty tests (see Fig. 10D for a schematic representation of these assays) (*49*). In the sociability task, while control mice spent more time exploring a stranger mouse (S1) compared to an inanimate object (O), both p110α^nKO^ and p110β^nKO^ spent similar times interacting with the mouse and the inanimate object; this was reflected in a reduction of the social preference index (Fig. 10, E and F). Next, in the social novelty test, control mice spent more time interacting with a stranger mouse (S2) than with the familiar one (S1). In contrast, both p110α^nKO^ and p110β^nKO^ mice displayed a lack of preference for interaction with either mouse (Fig. 10, G and H), which was more evident (and statistically different from control) in the case of p110β^nKO^. Therefore, these results suggest that removal of p110α or p110β impairs social behavior, both at the level of social preference and social memory.

## DISCUSSION

In this work, using a gene deletion approach in the hippocampus of adult mice, we have been able to ascribe specific functions to the major catalytic isoforms of type I PI3K, p110α, and p110β, with respect to neuronal structure, synaptic plasticity, and cognitive performance. This information sheds light into how PI3K activity mediates apparently antagonistic functions, such as mGluR-dependent synaptic depression and NMDAR-dependent synaptic potentiation. In addition, this study helps to clarify previously conflicting evidence for pre- or postsynaptic roles of PI3K during synaptic plasticity by establishing different loci of action for the different PI3K isoforms. We believe that this knowledge will be key to (i) understand the molecular mechanisms mediating the multiple actions of PI3K and (ii) define specific potential targets for pathological alterations of the PI3K/PTEN pathway, in cases such as autism, schizophrenia, or different forms of intellectual disability (*6*, *50*–*53*).

We show here that genetic deletion of p110α has a most profound effect disrupting neuronal and synaptic architecture. Using independent electrophysiological and morphological approaches, we report a marked decrease in neuron size, loss of dendritic complexity, and reduced number of synapses in p110α^nKO^ neurons. This combined loss of dendritic length and synapse number is likely responsible for the reduced basal transmission in these neurons and the loss of cytoskeletal and synaptic proteins observed in the proteomic analysis. It is interesting that the size of the remaining synapses in p110α^nKO^ neurons was significantly larger than in control neurons. This effect could be a consequence of a preferential loss of small synapses, or, alternatively, it may reflect a compensatory mechanism to preserve a certain level of synaptic drive in the face of a marked reduction in synapse number. The latter interpretation seems more likely, as the increase in synapse size was observed throughout the whole distribution of synapses, including large synapses [for example, the maximum area of the spine head, postsynaptic density (PSD) length, and PSD thickness all increased roughly by 50% in p110α^nKO^ neurons]. Incidentally, this result would also be consistent with the impairment we observed in NMDAR-dependent LTP since larger synapses in p110α^nKO^ neurons may be less plastic if they cannot be further potentiated. This interpretation is also backed-up by the increase in the levels of phosphorylated GluA1 receptors and cofilin. In addition, neurons were fully mature (1-month-old mice) when the genetic manipulation was executed. Therefore, these results imply that continuous PI3K activity provided by p110α is necessary for the maintenance of these dendritic and synaptic structures.

In the case of the p110β, we have found a dual role depending on the synaptic compartment. Thus, at the postsynaptic terminal, p110β contributes to NMDAR-dependent LTP, together with p110α. In addition, at the presynaptic terminal, p110β specifically limits glutamate release and mediates mGluR-LTD. These results fit previous observations linking increased p110β expression and enhanced mGluR-dependent LTD in the pathophysiology of Fragile X syndrome (*19*, *54*, *55*). Nevertheless, our results provide furthermechanistic information on mGluR-LTD and its locus of expression. Both presynaptic reduction in glutamate release (*56*–*60*) and postsynaptic removal of AMPARs (*61*–*63*) have been shown to occur during mGluR-LTD. Both types of mechanisms for synaptic depression are possibly concurrent, and their prevalence may change during postnatal development (*64*). It is also well established that PI3K activity is required for synaptic depression during mGluR-LTD (*17*–*19*). The insight we are providing here is that the involvement of PI3K in this form of plasticity is exclusively presynaptic (for CA3-to-CA1 synapses), is mediated by p110β, and is caused by a sustained decrease in neurotransmitter release. Further work will be required to determine how p110β regulates presynaptic glutamate release. This regulation may occur at the level of vesicle exocytosis. Thus, plasma membrane phosphatidylinositol 4,5-bisphosphate (PIP_2_) (the substrate for PIP_3_ synthesis by PI3K) has been shown to be essential for secretion in hippocampal neurons, and increasing levels of this phosphoinositide have been observed upon electrical stimulation (*65*). Therefore, p110β may inhibit neurotransmitter release by reducing PIP_2_ levels at the presynaptic membrane. Pharmacological inhibition of PI3K with LY294002 elicits a transient increase in both PIP_2_ and exocytosis in chromaffin cells, implying that controlling PIP_2_ levels on the plasma membrane could directly regulate exocytosis (*66*). On the other hand, p110β has previously been shown to promote autophagy (*67*) through its connection with the endocytic effector Rab5 (*68*). This mechanism may also link with presynaptic function, as neuronal autophagy reduces presynaptic release by limiting axonal endoplasmic reticulum calcium stores (*69*). A reduction in neuronal autophagy and protein turnover may also explain the accumulation of presynaptic vesicles (electron microscopy) and up-regulation of presynaptic proteins (proteomics) we have observed in the absence of p110β. Irrespective of the detailed mechanisms, these results have revealed a distinct molecular specialization of PI3K synaptic activities, with p110α in charge of postsynaptic structural maintenance and p110β limiting presynaptic vesicle release. To note, these specialized functions are described for excitatory CA3 to CA1 synapses. We cannot rule out that these PI3K isoforms play different roles in inhibitory neurons. Alternatively, it is also worth considering that similar molecular actions may result in different functional outcomes if the presynaptic and postsynaptic parameters of the neuron are different, as it is the case across multiple neuronal types.

Dysfunctions in neuronal communication are widely considered the underlying cause of many psychiatric and neurological diseases. The distinct but partially overlapping functions of p110α and p110β described here allow us to underscore specific cognitive alterations associated with different molecular and synaptic changes. Genetic deletion of either p110α or p110β isoform in the hippocampus produced hyperlocomotion (open field test). However, these phenotypes were qualitatively different with respect to the habituation component. Thus, p110β^nKO^ mice were not able to habituate to the novel environment in the time window we monitored. This lack of habituation may also be related to the failure to discriminate between novel and familiar objects in p110β^nKO^ mice (NORT). mGluR-LTD has been linked to sensory adaptation and novelty recognition (*70*), brain processes that are thought to be defective in neuropsychiatric disorders (*71*). We now propose that these deficits may be related to a failure to depress presynaptic function in the absence of p110β. In contrast, the hyperlocomotion and enhanced object exploration of p110α^nKO^ mice did not preclude habituation to the novel environment and conversely resulted in enhanced novel object discrimination. In a sense, it is remarkable that behavioral performance was fairly preserved in these memory tests (NOR and NOL) despite the profound reduction in synaptic connectivity observed in these animals. Other behavioral aspects were similarly impaired in p110α^nKO^ and p110β^nKO^, such as working memory (Y-maze test) and social interactions (both social recognition and social novelty). It is tempting to speculate that these deficits may be related to the impairment of LTP, which was observed in the absence of either p110α or p110β.

In summary, this work provides a genetic dissection of the molecular specialization of the PI3K pathway for the control of morphological and functional properties of neurons, as well as for proper synaptic communication in the mature brain. This specialization may be relevant for cognitive functions in the context of neurodevelopmental and psychiatric diseases and could pave the way for new therapeutic strategies in these neurological disorders.

## MATERIALS AND METHODS

### General experimental design

The objective of this study is to analyze the role of p110α and p110β in specific neuronal and cognitive functions. To this end, we have deleted the corresponding gene from p110α*^flox/flox^* or p110β*^flox/flox^* transgenic mice by expressing the *Cre* recombinase in hippocampal neurons of adult animals using AAVs. One month after infection, animals are subject to behavioral assays, and acute hippocampal slices are prepared for electrophysiological, biochemical, and microscopy experiments.

### Reagents

DL-AP5 and picrotoxin were purchased from Sigma-Aldrich. PI3K isoform–specific inhibitors A66, TGX221, and CAL101 were purchased form APExBIO. The antibody against p110α was generated in M.G.’s laboratory. Other primary antibodies were GluA1 (Abcam, ab31232), p110β (Abcam, ab151549), glyceraldehyde-3-phosphate dehydrogenase (Abcam, ab8245), pAKT(Thr^308^) (Cell Signaling Technology, 2965), pAKT(Ser^473^) (Cell Signaling Technology, 4060), tAKT (Cell Signaling Technology, 9272 and 2920), mGluR5 (Millipore, AB5675), actin (Millipore, MAB1501R), GluA2 (NeuroMab, 75-002), PSD95 (Thermo Fisher Scientific, MA1-046), mCherry (GeneTex, GTX59788), SAP102 (Stressgen, VAM-PS006), CaMKII (Sigma-Aldrich, c6974), GluN1 (Millipore, 05-432), GluN2A (Millipore, 07-632), and GluN2B (NeuroMab, 75-097).

### Proteomics

Three mice for each condition (C57/BL6, p110α*^lox/lox^*, and p110β*^lox/lox^*, infected in vivo with the AAV5-CaMKII-Cre-mCherry virus) were anesthetized and quickly decapitated once they were irresponsive to tail and foot pinches. The brains were rapidly removed and submerged in Ca^2+^-free ice-cold dissection solution [10 mM d-glucose, 4 mM KCl, 26 mM NaHCO_3_, 233.7 mM sucrose, 5 mM MgCl_2_, and 0.001% (w/v) phenol red as a pH indicator] previously saturated with carbogen (5% CO_2_ and 95% O_2_). Then, hippocampi were dissected and briefly washed on 1× ice-cold phosphate-buffered saline (137 mM NaCl, 2.68 mM KCl, 8.1 mM Na_2_HPO_4_, and 1.47 mM KH_2_PO_4_ with pH adjusted to 7.4) containing protease and phosphatase inhibitor cocktails (cOmplete Mini EDTA-free and phosSTOP, Roche) before being coarsely homogenized by cutting the tissue with sharp scissors, transferred to 2-ml vials prefilled with 200-μm zirconium beads (SPEX SamplePrep, 2302-200AW), and snap-frozen in dry ice. Hippocampus protein extracts samples were prepared in lysis buffer [50 mM tris-HCl–2% SDS (pH 8.5) and 10 mM tris-(2-carboxyethyl)phosphine] containing protease and phosphatase inhibitor cocktails (cOmplete Mini EDTA-free and phosSTOP, Roche). Peptides were quantified using TMT isobaric labeling, and labeled peptides were analyzed by liquid chromatography tandem mass spectrometry using a C-18 reversed-phase nano-column coupled to an Orbitrap Q Exactive HF mass spectrometer (Thermo Fisher Scientific) as described (*72*). For peptide identification, all spectra were analyzed with Proteome Discoverer (version 2.1.0.81, Thermo Fisher Scientific) using SEQUEST-HT. For database searching at the UniProt mouse database (September 2018; 53,780 entries), the parameters were selected as follows: trypsin digestion with two maximum missed cleavage sites, precursor and fragment mass tolerances of 2 and 0.02 Da, respectively, carbamidomethyl cysteine and TMT modifications at N terminus and Lys residues as fixed modifications, and methionine oxidation and Ser, Thr, and Tyr phosphorylation as dynamic modifications. Quantification of identified peptides was performed using the probability ratio method (*73*), and false discovery rate (FDR) was calculated using inverted databases and the refined method (*74*), with an additional filtering for a precursor mass tolerance of 15 parts per million (*75*). Statistical analysis of quantitative data for differential protein quantitation was performed with the in-house software SanXoT (*76*) using the WSPP statistical model previously described (*77*). In this model, protein and peptide log_2_ ratios are expressed as standardized variables, i.e., in units of SD according to their estimated variances (Zq values for protein quantification). Differences in protein abundance were estimated by the comparison of the compared groups’ Zq. A cutoff of ±1.2 or higher for Zq values and a *P* < 0.05 was used for the statistically significant identification of DEPs between the three experimental conditions. For functional analysis of the quantified proteins, identification of coordinated proteome changes and functional categories were performed using the Systems Biology Triangle algorithm (*78*) for the analysis of coordinated protein responses in high-throughput quantitative proteomics experiments. Posterior bioinformatic analysis was performed using the web-based tool G-profiler (Benjamini-Hochberg FDR correction threshold of 0.01) and RStudio with custom-made scripts using the packages: PCAtools v 2.1.22, pheatmap v 1.0.12, and corrplot v0.84. Principal components analysis and unsupervised hierarchical clustering from the total proteome were used to visualize sample clustering and variability.

### Animals and ethics statement

For the organotypic hippocampal cultured slices, we used Wistar rats of both genders. These animals were housed in standard cages with ad libitum access to food and water under a 12:12 hour dark-light cycle in humidity- and temperature-controlled rooms. The rest of the experiments were performed using adult mice (6 to 8 months). All mice used were housed in standard cages (maximum of five animals per cage) in humidity- and temperature-controlled rooms. Autoventilated racks contained individually ventilated animal cages with absolute filters. Irradiated standard safe diet and autoclaved water were available ad libitum.

p110α*^flox/flox^* is a transgenic mouse line with the 18th and 19th exons of the *PIK3CA* gene flanked by two LoxP sequences. p110β*^flox/flox^* is a transgenic mouse line with the 21st and 22nd exons of the *PIK3CB* gene flanked by two LoxP sequences. These mice were contributed by M.G.’s laboratory. Transgenesis procedures to generate these mice were previously described (*39*, *79*). Their genetic background is C57BL/6J (the Jackson Laboratory). Genotyping was carried out by polymerase chain reaction using the following primers: p110α*^flox/flox^*, GGATGCGGTCTTTATTGTC (FE1) and TGGCATGCTGCCGAATTG (FE4); p110β*^flox/flox^*, AGTGAACGCTATGCATCACACCAGC (b3) and AAGTACAAACATCCAAGCAA (b98). All biosafety procedures and animal experimental protocols were approved by the Ethical Committee from the Consejo Superior de Investigaciones Científicas, in strict accordance with Spanish (RD 53/2013, 32/2007) and E.U. guidelines set out in the European Community Council Directives (2010/63/EU and 86/609/EEC).

### Stereotaxic in vivo microinjections of AAV and Sindbis virus

AAV5-CaMKII-Cre-mCherry virus (serotype 5, 5.8 × 10^12^ viral particles/ml, Gene Therapy Vector Core at the University of North Carolina) was delivered to the hippocampus through two bilateral injections (650 nl each, 120 nl/min) in the dorsal [from bregma: anterior-posterior (AP), −1.8 mm; medial-lateral (ML), ±1.3 mm; dorsal-ventral (DV), −1.7 mm] and ventral (AP, −2.8 mm; ML, ±2.4 mm; DV, −2 mm) regions. The 3- to 4-month-old animals were placed in the stereotaxic frame (Harvard Apparatus) over a heating pad and immobilized using blunt ear holders. Anesthesia was initiated and maintained by inhalation of 2% isoflurane in oxygen. Viral solution was infused using a Hamilton Neuros syringe (65460-02) inserted through small holes drilled into the skull and coupled to a micropump (120 nl/min; Quintessential Stereotaxic Injector, Stoelting, 53311). After the infusion, the syringe was kept in place for 5 min and slowly withdrawn. After the closure of the wound, the animals received analgesia by subcutaneous injection of meloxicam (5 mg/kg of dose). For CA1-specific injections, we used the same protocol, but we performed three bilateral injections (50 nl each, 50 nl/min): rostral (AP, −1.8 mm; ML, ±1.3 mm; DV, −1.6 mm), medial (AP, −2.3 mm; ML, ±1.7 mm; DV, −1.7 mm), and caudal (AP, −2.8 mm; ML, ±2.6 mm; DV, −2 mm). The accuracy of the infection was confirmed after 1 month by immunofluorescence using the expression of mCherry as the reporter. For neuron morphology experiments, injected mice were subjected to a second round of in vivo injections using the previous parameters to inject Sindbis viruses expressing GFP. Four weeks after infection, we observed Cre-expressing cells, which were distributed along the entire hippocampus. The rate of infection, as evaluated by colocalization with neuronal markers was 89 ± 5.2% (*N* = 5) (*38*).

### Behavior

#### Open field test

This test was used to evaluate general locomotor activity and as a habituation phase to NOLT and NORT. Mice were placed individually in the center of an acrylic box (40 cm by 40 cm by 40 cm) with white opaque walls at ground level. The animals were allowed to explore the arena for 5 min while being recorded. Their behavior was tracked using ANY-maze software.

#### NOLT, NORT, and reexposure test

Assays were performed on 3- to 4-month-old mice 24 hours after the open field test, which was used as habituation phase for NOLT, NORT, and reexposure. In the first phase of the NORT, mice were placed in the open field arena containing two identical objects in terms of color, texture, and shape on two adjacent corners of the arena. Mice were allowed to explore the objects for 7 min while being recorded. In the test phase of the NORT (3 hours later), one of the objects was switched for another one of similar dimensions and texture but different in color and shape. Then, mice were allowed to explore both objects for 7 min. The objects used were a pair of orange triangular prisms and a pair of blue pyramids. Both groups of objects were randomly assigned as familiar or novel among the subjects to ensure that they showed no preference for any of them. In the NOLT, the objects were a pair of white square prisms identical in shape, dimensions, and texture. During the familiarization phase, both objects were placed in two adjacent corners. In the test phase of the NOLT, the location of one of the objects was changed to the opposite corner. In the reexposure testing, after habituating in an empty box on day 1, mice were exposed to two novel objects on the following day (training) and then reexposed to the same objects 3 hours later (reexposure). Data were expressed as a percentage of reexploration (“reexposure”) relative to the initial exploration time (during “training”). In the NORT, NOLT, and reexposure, the arena and the objects were cleaned thoroughly with 70% ethanol in between phases to eliminate any olfactory cues. Behavior was recorded from a camera located above the arena, and the analysis was performed manually in time segments of 1 min by an experimenter blinded to the treatment using the ANY-maze software. Exploration of the objects was defined as close contact sniffing (with the head pointed toward the object and within 2 cm of the object). We then calculated a discrimination index as the time spent exploring the relocated or novel object divided by the time exploring both objects.

#### Social interaction and social memory test

This task is used to evaluate social interaction in mice and their preference to establish social contact. The social memory task also relies on the mice’s innate preference for novelty. For this task, we used a three-chamber acrylic box with clear dividing walls that have a hole to give the testing mouse access to each chamber. This test consisted of three phases. On the first phase, wire cages were placed on the two outermost chambers allowing visual, auditory, olfactory, and minimal touch interaction. Mice were allowed to explore the arena for 5 min. For the second phase, the social interaction task was performed 24 hours later. An unfamiliar mouse was placed under the wire cage of one of the chambers (gender- and age-paired), and an inanimate object was placed on the opposite cage in an identical configuration. The unfamiliar mice had been habituated to the wire cage for intervals of 15 min before the test. In the testing phase, the subject mouse was placed in the center chamber and allowed to freely explore all three chambers for 7 min. The test mouse was removed, and the third phase was immediately started after cleaning the arena and the wire cages thoroughly with ethanol 70% to eliminate any olfactory cues. For the third phase, the social memory task, the inanimate object was replaced by another unfamiliar mouse, and the test was performed exactly as in the second phase. Behavior was recorded from a camera located above the test box, and analysis was performed manually in time segments of 1 min. We measured number of visits to each chamber, time spent on each chamber, and exploration. Exploration of the targets was defined as close contact sniffing of the wire cage (with the head pointed toward the cage). Sitting or standing time on the wire cages was not quantified as exploration. We also calculated the discrimination index as the time exploring the subject versus the time exploring the subject and the object in the first phase and the time exploring the new subject versus the new and the familiar.

#### Elevated plus maze test

This test is widely accepted to test anxiety-like behavior (*80*). The apparatus consists of a plus-shaped maze elevated 45 cm from the ground and has two opposite positioned open arms with railing (5 cm by 29.5 cm by 0.3 cm), two opposite closed arms (5 cm by 29.5 cm by 15 cm), and a center area (5 cm by 5 cm). Mice were tested in a single trial by being placed in the center area and recorded from above for 5 min. To eliminate odor cues, the maze was thoroughly cleaned with 70% ethanol and allowed to dry in between subjects. The analysis was performed in time segments of 1 min both manually and using the ANY-maze software.

#### Y-maze test

The Y-maze test was performed to quantify spontaneous alternation and assess deficits or enhancements on spatial working memory. The apparatus consists of a Y-shaped maze with three identical enclosed arms. Each arm was assigned a letter from A to C. To perform the test, we placed each mouse in the center of the maze and recorded a single trial of 5 min from above. To eliminate odor cues, the maze was thoroughly cleaned with 70% ethanol and allowed to dry in between subjects. The sequence of arm entrance was recorded manually, and an alternation is considered a consecutive entrance in all three arms. The spontaneous alternation percentage was calculated using the following equation: % spontaneous alternation = (number of alternations) × 100 / (total number of arm entrances − 2).

### Hippocampal slice preparation

#### Organotypic cultured hippocampal slices

Organotypic cultures were established from rat pups (5 to 7 days old) according to previously described procedures. Animals were anesthetized and quickly decapitated once they were irresponsive to tail and foot pinches. Whole brains were extracted and immersed in ice-cold dissection solution [10 mM glucose, 4 mM KCl, 26 mM NaHCO_3_, 233.7 mM sucrose, 5 mM MgCl_2_, and 1 mM CaCl_2_ with 0.001% (w/v) phenol red as a pH indicator], previously saturated with carbogen (5% CO_2_ and 95% O_2_). Hippocampi were dissected under sterile conditions and placed over a tissue slicer (Stoelting Europe, 51425) to obtain 400-μm-thick slices. These slices were then transferred to porous nitrocellulose membranes (Merck Millipore, PICM0RG50) on culture medium [0.8% (w/v) minimum essential medium powder, 20% (v/v) horse serum, 1 mM l-glutamine, 1 mM CaCl_2_, 2 mM MgSO_4_, insulin (1 mg/liter), 0.0012% (v/v) ascorbic acid, 30 mM Hepes, 13 mM d-glucose, and 5.2 mM NaHCO_3_] and maintained at 35.5°C and 5% CO_2_ for a minimum of 7 days and up to 15 days. The culture medium was replaced every 2 to 3 days. These slices were recorded in artificial cerebrospinal fluid (aCSF) containing 119 mM NaCl, 2.5 mM KCl, 1 mM NaH_2_PO_4_, 11 mM glucose, 26 mM NaHCO_3_, 4 mM MgCl_2_, 4 mM CaCl_2_, and osmolarity adjusted to 290 ± 5 mOsm. For electrophysiological experiments in the presence of pharmacological inhibitors, 0.01% dimethyl sulfoxide (DMSO; vehicle), 2.5 μM A66, or 0.5 μM TGX221 in 0.01% DMSO were added to the culture medium for overnight treatment and reapplied 2 hours before the experiment. These inhibitors were also added to the aCSF during the recordings at the same concentrations. The identity of the pharmacological inhibitors was coded so that the experiments could be performed blindly.

#### Acute slices for field recordings

Acute hippocampal slices were obtained from 4-month-old mice of both genders. Animals were anesthetized and quickly decapitated once they were irresponsive to tail and foot pinches. The brains were rapidly removed and submerged in Ca^2+^-free ice-cold dissection solution (10 mM d-glucose, 4 mM KCl, 26 mM NaHCO_3_, 233.7 mM sucrose, 5 mM MgCl_2_, and 0.001% (w/v) phenol red as a pH indicator) previously saturated with carbogen (5% CO_2_ and 95% O_2_). Coronal slices (350 μm) were obtained by cutting the brain in the same solution with a vibratome (Leica, VT1200S) and left in carbogen-gassed aCSF (119 mM NaCl, 2.5 mM KCl, 1 mM NaH_2_PO_4_, 26 mM NaHCO_3_, 11 mM glucose, 1.2 mM MgCl_2_, 2.5 mM CaCl_2_, and osmolarity adjusted to 290 mOsm) for 1 hour at 32°C to recover. Past that time, the slices were maintained at 25°C, and the recordings were performed in the same aCSF solution used for recovery.

#### Acute slices for whole-cell recordings

Slices were obtained as previously described (*38*). Briefly, animals were anesthetized, and once they became irresponsive to foot and tail pinches, they were intracardially perfused with cold-choline aCSF (92 mM choline chloride, 2.5 mM KCl, 1.2 mM NaH_2_PO_4_, 30 mM NaHCO_3_, 20 mM Hepes, 25 mM glucose, 5 mM sodium ascorbate, 2 mM thiourea, 3 mM sodium pyruvate, 10 mM MgSO_4_, 0.5 mM CaCl_2_, and osmolarity adjusted to 305 ± 5 mOsm and pH to 7.35). The brains were rapidly removed and placed in cold-choline aCSF. Coronal slices (350 μm) were obtained by cutting the brain in cold-choline aCSF. Then, the slices were recovered for <10 min in warm-choline aCSF (32°C) and then transferred to Hepes holding aCSF (92 mM NaCl, 2.5 mM KCl, 1.2 mM NaH_2_PO_4_, 30 mM NaHCO_3_, 20 mM Hepes, 25 mM glucose, 5 mM sodium ascorbate, 2 mM thiourea, 3 mM sodium pyruvate, 2 mM MgSO_4_, 2 mM CaCl_2_, and osmolarity adjusted to 305 ± 5 mOsm and pH to 7.35) for 1 hour at 32°C. Past that time, the slices were maintained in recording aCSF (119 mM NaCl, 2.5 mM KCl, 1.2 mM NaH_2_PO_4_, 24 mM NaHCO_3_, 12.5 mM glucose, 2 mM MgSO_4_, 2 mM CaCl_2_, osmolarity adjusted to 305 ± 5 mOsm and pH to 7.35) at 30°C, and the electrophysiological recordings were performed in the same solution.

### Electrophysiology

Excitatory postsynaptic currents (EPSCs) and fEPSPs were recorded from CA1 pyramidal neurons with glass recording electrodes while stimulating Schaffer collateral fibers to evoke synaptic responses. During the recordings, the slices were placed in an immersion chamber constantly perfused with aCSF. The aCSF was continuously gassed with 5% CO_2_ and 95% O_2_, and its temperature was closely monitored and maintained at 29°C for whole-cell recordings and at 25°C for all field recording experiments except for mGluR-LTD experiments, which were performed at 30°C. For all experiments, aCSF was supplemented with 100 μM picrotoxin to block γ-aminobutyric acid type A receptors. For mGluR-LTD, 100 μM DL-AP5 was added to the aCSF to block NMDARs. Patch-clamp recording glass electrodes (4 to 8 MOhm) were composed of silver/silver chloride electrodes inserted in glass pipettes filled with intracellular solution (115 mM CsMeSO_3_, 20 mM CsCl, 10 mM Hepes, 2.5 mM MgCl_2_, 4 mM Na_2_-ATP, 0.4 mM Na-guanosine triphosphate, 10 mM sodium phosphocreatine, 0.6 mM EGTA, 10 mM lidocaine *N*-ethyl bromide, and pH adjusted to 7.25 and osmolarity to 290 ± 5 mOsm). In the case of field recordings, glass pipettes (0.5 to 1 MOhm) were filled with the aCSF used as extracellular solution and placed in CA1 stratum radiatum.

AMPAR-mediated responses were measured at −60 mV. NMDAR-mediated responses were measured at +40 mV at a point when AMPAR-mediated responses had fully decayed (65 ms after stimulation). PPR experiments were performed with 50-ms interstimulus intervals. NMDAR-dependent LTP under whole-cell patch clamp was induced using a pairing protocol that consists of presynaptic stimulation at 3 Hz (300 pulses) coupled to postsynaptic depolarization to 0 mV. NMDAR-dependent LTP for field recordings was induced using a theta-burst protocol composed of 10 trains of bursts (four pulses at 100 Hz with a 200-ms interval), and it was repeated for four cycles with 20-s intercycle interval. NMDAR-dependent LTD was induced using low-frequency (1 Hz, 900 pulses) presynaptic stimulation. mGluR-dependent LTD was induced using low-frequency (1 Hz, 900 pulses) presynaptic stimulation with paired pulses (50-ms interstimulus interval). Stimulation intensity was adjusted to 30% (for LTP and PPR) or 70% (for LTD) of the maximum response.

Data acquisition was carried out with MultiClamp 700 A/B amplifiers and pClamp software (Molecular Devices). Data analysis was performed using custom-made Excel (Microsoft) macros.

### Confocal fluorescence microscopy and morphometry

Mice were anesthetized by isoflurane inhalation and intracardially perfused with phosphate buffer (PB) to wash the vascular system before injecting the fixative [4% paraformaldehyde in 0.1 M PB (pH 7.4)]. Brains were postfixed overnight by immersion in the same fixative solution at 4°C and then sequentially dehydrated in solutions containing 15 and 30% sucrose in PB at 4°C. Brains were cut in 100-μm coronal slices, stained with 4′,6-diamidino-2-phenylindole (DAPI; 1 μg/ml) for 5 min and mounted on adherent microscope slides (Thermo Fisher Scientific, 15438060) with ProLong Glass Antifade (Thermo Fisher Scientific, P-36982). Fluorescence images for dendrite morphology were acquired as Z-stacks with a confocal inverted microscope (Zeiss, LSM800) using a 25× numerical aperture (NA) 0.8 Plan-Apochromat M27 oil immersion objective and 488- and 555-nm lasers. Z-stacks were reconstructed using Fiji v1.51 (average intensity projection), and the neuronal dendritic trees were two-dimensionally traced with the simple neurite tracer plug-in. The Sholl analysis was performed counting the number of intersections in 10-μm intervals from the soma as well as the total basal and apical dendritic length. Images for spine morphology were acquired as Z-stacks using a 63× NA 0.8 Plan-Apochromat M27 oil immersion objective and a 488-nm laser. For deconvolution purposes, spine morphology images were acquired under an optimal calculated zoom of 3×, an XY pixel size of <43 nm, and a Z-step size of 130 nm. The stacks were processed after image acquisition with a three-dimensional deconvolution algorithm using Huygens software (Huygens 19.10, SVI). Spine density was defined as the number of spines divided by the corresponding dendritic length.

### Electron microscopy

Mice were anesthetized by isoflurane inhalation and intracardiacally perfused with PB to wash the vascular system before injecting the fixative [4% paraformaldehyde and 2% glutaraldehyde in 0.1 M PB (pH 7.4)]. Brains were left for 2 hours at room temperature and overnight at 4°C immersed in the same fixative solution. Coronal slices of 200 μm were obtained and postfixed in a 2% solution of osmium tetroxide in 0.1 M PB (1.5 hours at room temperature). The slices were then washed and stained with 2% uranyl acetate in water (1 hour at room temperature in darkness), washed, dehydrated in increasing concentrations of ethanol, and embedded in Epon resin. Series of ultrathin sections of the CA1 region of the hippocampus were collected and mounted on single oval slot copper/palladium grids. The sections were imaged on a JEM-1400Flash transmission electron microscope (JEOL) coupled to a complementary metal-oxide semiconductor Oneview camera (Gatan). The stratum radiatum proximal to the stratum pyramidale was imaged with a ×8000 magnification, and the PSD size, length, and number of vesicles were measured manually using Fiji v1.51.

### Western blotting

Brains were extracted from four animals for each condition (control, p110α^nKO^, and p110β^nKO^). Hippocampi were dissected and homogenized in a glass-teflon potter on ice-cold buffer containing 20 mM Hepes, 0.1% NP-40, cOmplete mini EDTA-free, and phosSTOP and centrifuged at 13,000*g* for 10 min at 4°C. The supernatant processed on SDS–polyacrylamide gel electrophoresis and transferred to a polyvinylidene difluoride membrane (Immoblot-P Millipore). After membrane blocking for 1 hour in 5% skim milk in Tris-buffered saline with 0.1% Tween 20, primary antibodies were incubated overnight at 4°C in blocking solution. Membranes were washed in TBST and incubated for 1 hour in the corresponding secondary antibodies (peroxidase-conjugated anti-rabbit or anti-mouse; the Jackson ImmunoResearch, 711-035-152 and 715-035-151). Detection was carried out by chemiluminescence (Immobilon Western, Millipore) using ImageQuant LAS 4000 mini biomolecular imager.

### Statistical analyses

Unless otherwise indicated, results were represented as means ± SEM. For pairwise comparisons, *P* values were calculated according to two-tailed Mann-Whitney tests (for unpaired data) or Wilcoxon tests (for paired data). For multiple comparisons, we used two-way repeated-measures analysis of variance (ANOVA), followed by Bonferroni’s post hoc analysis. The values for *N*, *P*, and the specific statistical test performed for each experiment are described in the corresponding figure legend. A complete report of the statistical analysis of the proteomics data can be found above in the “Proteomics” section and in data S1.

## Notes

View/request a protocol for this paper from *Bio-protocol*.

## References

[1] T. V. P. Bliss, G. L. Collingridge,A synaptic model of memory: Long-term potentiation in the hippocampus. Nature 361,31–39 (1993). 8421494 10.1038/361031a0

[2] S. J. Martin, P. D. Grimwood, R. G. M. Morris,Synaptic plasticity and memory: An evaluation of the hypothesis. Annu. Rev. Neurosci. 23,649–711 (2000). 10845078 10.1146/annurev.neuro.23.1.649

[3] S. Knafo, J. A. Esteban,Common pathways for growth and for plasticity. Curr. Opin. Neurobiol. 22,405–411 (2012). 22398399 10.1016/j.conb.2012.02.008

[4] K. L. Arendt, M. Royo, M. Fernández-Monreal, S. Knafo, C. N. Petrok, J. R. Martens, J. A. Esteban,PIP 3 controls synaptic function by maintaining AMPA receptor clustering at the postsynaptic membrane. Nat. Neurosci. 13,36–44 (2010). 20010819 10.1038/nn.2462 PMC2810846

[5] S. Jurado, M. Benoist, A. Lario, S. Knafo, C. N. Petrok, J. A. Esteban,PTEN is recruited to the postsynaptic terminal for NMDA receptor-dependent long-term depression. EMBO J. 29,2827–2840 (2010). 20628354 10.1038/emboj.2010.160 PMC2924645

[6] S. Knafo, J. A. Esteban,PTEN: Local and global modulation of neuronal function in health and disease. Trends Neurosci. 40,83–91 (2017). 28081942 10.1016/j.tins.2016.11.008

[7] C. Sánchez-Puelles, M. Calleja-Felipe, A. Ouro, G. Bougamra, A. Arroyo, I. Diez, A. Erramuzpe, J. Cortés, J. Martínez-Hernández, R. Luján, M. Navarrete, C. Venero, A. Chan, M. Morales, J. A. Esteban, S. Knafo,PTEN activity defines an axis for plasticity at cortico-amygdala synapses and influences social behavior. Cereb. Cortex 30,505–524 (2020). 31240311 10.1093/cercor/bhz103

[8] K. Takeuchi, M. J. Gertner, J. Zhou, L. F. Parada, M. V. L. Bennett, R. S. Zukin,Dysregulation of synaptic plasticity precedes appearance of morphological defects in a Pten conditional knockout mouse model of autism. Proc. Natl. Acad. Sci. U.S.A. 110,4738–4743 (2013). 23487788 10.1073/pnas.1222803110 PMC3607034

[9] P. J. Blair, J. Harvey,PTEN: A new player controlling structural and functional synaptic plasticity. J. Physiol. 590,1017–1017 (2012). 22399818 10.1113/jphysiol.2012.227868 PMC3381807

[10] M. M. Fraser, I. T. Bayazitov, S. S. Zakharenko, S. J. Baker,Phosphatase and tensin homolog, deleted on chromosome 10 deficiency in brain causes defects in synaptic structure, transmission and plasticity, and myelination abnormalities. Neuroscience 151,476–488 (2008). 18082964 10.1016/j.neuroscience.2007.10.048 PMC2278004

[11] D. K. Chow, M. Groszer, M. Pribadi, M. Machniki, S. T. Carmichael, X. Liu, J. T. Trachtenberg,Laminar and compartmental regulation of dendritic growth in mature cortex. Nat. Neurosci. 12,116–118 (2009). 19151711 10.1038/nn.2255 PMC2842592

[12] K. L. Arendt, M. Royo, M. Fernández-Monreal, S. Knafo, C. N. Petrok, J. R. Martens, J. A. Esteban,PIP3 controls synaptic function by maintaining AMPA receptor clustering at the postsynaptic membrane. Nat. Neurosci. 13,36–44 (2010). 20010819 10.1038/nn.2462 PMC2810846

[13] P. Opazo, A. M. Watabe, S. G. N. Grant, T. J. O’Dell,Phosphatidylinositol 3-kinase regulates the induction of long-term potentiation through extracellular signal-related kinase-independent mechanisms. J. Neurosci. 23,3679–3688 (2003). 12736339 10.1523/JNEUROSCI.23-09-03679.2003 PMC6742185

[14] P. P. Sanna, M. Cammalleri, F. Berton, C. Simpson, R. Lutjens, F. E. Bloom, W. Francesconi,Phosphatidylinositol 3-kinase is required for the expression but not for the induction or the maintenance of long-term potentiation in the hippocampal CA1 region. J. Neurosci. 22,3359–3365 (2002). 11978812 10.1523/JNEUROSCI.22-09-03359.2002 PMC6758361

[15] H. Y. Man, Q. Wang, W. Y. Lu, W. Ju, G. Ahmadian, L. Liu, S. D’Souza, T. P. Wong, C. Taghibiglou, J. Lu, L. E. Becker, L. Pei, F. Liu, M. P. Wymann, J. F. MacDonald, Y. T. Wang,Activation of PI3-kinase is required for AMPA receptor insertion during LTP of mEPSCs in cultured hippocampal neurons. Neuron 38,611–624 (2003). 12765612 10.1016/s0896-6273(03)00228-9

[16] C. H. Lin, S. H. Yeh, C. H. Lin, K. T. Lu, T. H. Leu, W. C. Chang, P. W. Gean,A role for the PI-3 kinase signaling pathway in fear conditioning and synaptic plasticity in the amygdala. Neuron 31,841–851 (2001). 11567621 10.1016/s0896-6273(01)00433-0

[17] L. Hou, E. Klann,Activation of the phosphoinositide 3-kinase-Akt-mammalian target of rapamycin signaling pathway is required for metabotropic glutamate receptor-dependent long-term depression. J. Neurosci. 24,6352–6361 (2004). 15254091 10.1523/JNEUROSCI.0995-04.2004 PMC6729543

[18] W. B. Potter, T. Basu, K. J. O’Riordan, A. Kirchner, P. Rutecki, C. Burger, A. Roopra,Reduced juvenile long-term depression in tuberous sclerosis complex is mitigated in adults by compensatory recruitment of mGluR5 and Erk signaling. PLOS Biol. 11,e1001627 (2013). 23966835 10.1371/journal.pbio.1001627 PMC3742461

[19] C. Gross, A. Banerjee, D. Tiwari, F. Longo, A. R. White, A. G. Allen, L. M. Schroeder-Carter, J. C. Krzeski, N. A. Elsayed, R. Puckett, E. Klann, R. A. Rivero, S. L. Gourley, G. J. Bassell,Isoform-selective phosphoinositide 3-kinase inhibition ameliorates a broad range of fragile X syndrome-associated deficits in a mouse model. Neuropsychopharmacology 44,324–333 (2019). 30061744 10.1038/s41386-018-0150-5 PMC6300538

[20] J. I. Kim, H. R. Lee, S. E. Sim, J. Baek, N. K. Yu, J. H. Choi, H. G. Ko, Y. S. Lee, S. W. Park, C. Kwak, S. J. Ahn, S. Y. Choi, H. Kim, K. H. Kim, P. H. Backx, C. A. Bradley, E. Kim, D. J. Jang, K. Lee, S. J. Kim, M. Zhuo, G. L. Collingridge, B. K. Kaang,PI3Kγ is required for NMDA receptor-dependent long-term depression and behavioral flexibility. Nat. Neurosci. 14,1447–1454 (2011). 22019731 10.1038/nn.2937

[21] J. H. Choi, P. Park, G. C. Baek, S. E. Sim, S. J. J. Kang, Y. Lee, S. H. Ahn, C. S. Lim, Y. S. Lee, G. L. Collingridge, B. K. Kaang,Effects of PI3Kγ overexpression in the hippocampus on synaptic plasticity and spatial learning. Mol. Brain 7,78 (2014). 25373491 10.1186/s13041-014-0078-6 PMC4226891

[22] V. L. Hood, C. Paterson, A. J. Law,PI3Kinase-p110δ overexpression impairs dendritic morphogenesis and increases dendritic spine density. Front. Mol. Neurosci. 13,29 (2020). 32180704 10.3389/fnmol.2020.00029 PMC7059765

[23] J. Jaworski, S. Spangler, D. P. Seeburg, C. C. Hoogenraad, M. Sheng,Control of dendritic arborization by the phosphoinositide-3′-kinase-Akt-mammalian target of rapamycin pathway. J. Neurosci. 25,11300–11312 (2005). 16339025 10.1523/JNEUROSCI.2270-05.2005 PMC6725892

[24] V. Kumar, M. X. Zhang, M. W. Swank, J. Kunz, G. Y. Wu,Regulation of dendritic morphogenesis by Ras-PI3K-Akt-mTOR and Ras-MAPK signaling pathways. J. Neurosci. 25,11288–11299 (2005). 16339024 10.1523/JNEUROSCI.2284-05.2005 PMC6725910

[25] A. Martín-Peña, A. Acebes, J. R. Rodríguez, A. Sorribes, G. G. De Polavieja, P. Fernández-Fúnez, A. Ferrús,Age-independent synaptogenesis by phosphoinositide 3 kinase. J. Neurosci. 26,10199–10208 (2006). 17021175 10.1523/JNEUROSCI.1223-06.2006 PMC6674615

[26] G. Cuesto, L. Enriquez-Barreto, C. Caramés, M. Cantarero, X. Gasull, C. Sandi, A. Ferrús, Á. Acebes, M. Morales,Phosphoinositide-3-kinase activation controls synaptogenesis and spinogenesis in hippocampal neurons. J. Neurosci. 31,2721–2733 (2011). 21414895 10.1523/JNEUROSCI.4477-10.2011 PMC6623769

[27] S. Jordán-Álvarez, W. Fouquet, S. J. Sigrist, A. Acebes,Presynaptic PI3K activity triggers the formation of glutamate receptors at neuromuscular terminals of *Drosophila*. J. Cell Sci. 125,3621–3629 (2012). 22505608 10.1242/jcs.102806

[28] S. Jordán-Álvarez, E. Santana, S. Casas-Tintó, Á. Acebes, A. Ferrús,The equilibrium between antagonistic signaling pathways determines the number of synapses in *Drosophila*. PLOS ONE 12,3621–3629 (2017). 10.1371/journal.pone.0184238 PMC5593197 28892511

[29] K. E. Cosker, B. J. Eickholt,Phosphoinositide 3-kinase signalling events controlling axonal morphogenesis. Biochem. Soc. Trans. 35,207–210 (2007). 17371239 10.1042/BST0350207

[30] L. C. Cantley,The phosphoinositide 3-kinase pathway. Science 296,1655–1657 (2002). 12040186 10.1126/science.296.5573.1655

[31] B. Bilanges, Y. Posor, B. Vanhaesebroeck,PI3K isoforms in cell signalling and vesicle trafficking. Nat. Rev. Mol. Cell Biol. 20,515–534 (2019). 31110302 10.1038/s41580-019-0129-z

[32] B. Vanhaesebroeck, J. Guillermet-Guibert, M. Graupera, B. Bilanges,The emerging mechanisms of isoform-specific PI3K signalling. Nat. Rev. Mol. Cell Biol. 11,329–341 (2010). 20379207 10.1038/nrm2882

[33] B. Vanhaesebroeck, M. J. Welham, K. Kotani, R. Stein, P. H. Warne, M. J. Zvelebil, K. Higashi, S. Volinia, J. Downward, M. D. Waterfield,p110δ, A novel phosphoinositide 3-kinase in leukocytes. Proc. Natl. Acad. Sci. U.S.A. 94,4330–4335 (1997). 9113989 10.1073/pnas.94.9.4330 PMC20722

[34] I. Cuscó, A. Medrano, B. Gener, M. Vilardell, F. Gallastegui, O. Villa, E. González, B. Rodríguez-Santiago, E. Vilella, M. Del Campo, L. A. Pérez-Jurado,Autism-specific copy number variants further implicate the phosphatidylinositol signaling pathway and the glutamatergic synapse in the etiology of the disorder. Hum. Mol. Genet. 18,1795–1804 (2009). 19246517 10.1093/hmg/ddp092 PMC2671988

[35] A. J. Law, Y. Wang, Y. Sei, P. O’Donnell, P. Piantadosi, F. Papaleo, R. E. Straub, W. Huang, C. J. Thomas, R. Vakkalanka, A. D. Besterman, B. K. Lipska, T. M. Hyde, P. J. Harrison, J. E. Kleinman, D. R. Weinberger,Neuregulin 1-ErbB4-PI3K signaling in schizophrenia and phosphoinositide 3-kinase-p110δ inhibition as a potential therapeutic strategy. Proc. Natl. Acad. Sci. U.S.A. 109,12165–12170 (2012). 22689948 10.1073/pnas.1206118109 PMC3409795

[36] L. Bi, I. Okabe, D. J. Bernard, A. Wynshaw-Boris, R. L. Nussbaum,Proliferative defect and embryonic lethality in mice homozygous for a deletion in the p110α subunit of phosphoinositide 3-kinase. J. Biol. Chem. 274,10963–10968 (1999). 10196176 10.1074/jbc.274.16.10963

[37] L. Bi, I. Okabe, D. J. Bernard, R. L. Nussbaum,Early embryonic lethality in mice deficient in the p110β catalytic subunit of PI 3-kinase. Mamm. Genome 13,169–172 (2002). 11919689 10.1007/BF02684023

[38] M. Navarrete, M. I. Cuartero, R. Palenzuela, J. E. Draffin, A. Konomi, I. Serra, S. Colié, S. Castaño-Castaño, M. T. Hasan, Á. R. Nebreda, J. A. Esteban,Astrocytic p38α MAPK drives NMDA receptor-dependent long-term depression and modulates long-term memory. Nat. Commun. 10,2968 (2019). 31273206 10.1038/s41467-019-10830-9 PMC6609681

[39] M. Graupera, J. Guillermet-Guibert, L. C. Foukas, L. K. Phng, R. J. Cain, A. Salpekar, W. Pearce, S. Meek, J. Millan, P. R. Cutillas, A. J. H. Smith, A. J. Ridley, C. Ruhrberg, H. Gerhardt, B. Vanhaesebroeck,Angiogenesis selectively requires the p110α isoform of PI3K to control endothelial cell migration. Nature 453,662–666 (2008). 18449193 10.1038/nature06892

[40] S. Xie, J. Ni, J. R. McFaline-Figueroa, Y. Wang, R. T. Bronson, K. L. Ligon, P. Y. Wen, T. M. Roberts, J. J. Zhao,Divergent roles of PI3K isoforms in PTEN-deficient glioblastomas. Cell Rep. 32,108196 (2020). 32997991 10.1016/j.celrep.2020.108196 PMC7571617

[41] P. S. Kaeser, W. G. Regehr,The readily releasable pool of synaptic vesicles. Curr. Opin. Neurobiol. 43,63–70 (2017). 28103533 10.1016/j.conb.2016.12.012 PMC5447466

[42] S. Q. Ren, J. Z. Yan, X. Y. Zhang, Y. F. Bu, W. W. Pan, W. Yao, T. Tian, W. Lu,PKCλ is critical in AMPA receptor phosphorylation and synaptic incorporation during LTP. EMBO J. 32,1365–1380 (2013). 23511975 10.1038/emboj.2013.60 PMC3655466

[43] S. P. Jackson, S. M. Schoenwaelder, I. Goncalves, W. S. Nesbitt, C. L. Yap, C. E. Wright, V. Kenche, K. E. Anderson, S. M. Dopheide, Y. Yuan, S. A. Sturgeon, H. Prabaharan, P. E. Thompson, G. D. Smith, P. R. Shepherd, N. Daniele, S. Kulkarni, B. Abbott, D. Saylik, C. Jones, L. Lu, S. Giuliano, S. C. Hughan, J. A. Angus, A. D. Robertson, H. H. Salem,PI 3-kinase p110β: A new target for antithrombotic therapy. Nat. Med. 11,507–514 (2005). 15834429 10.1038/nm1232

[44] S. Jamieson, J. U. Flanagan, S. Kolekar, C. Buchanan, J. D. Kendall, W. J. Lee, G. W. Rewcastle, W. A. Denny, R. Singh, J. Dickson, B. C. Baguley, P. R. Shepherd,A drug targeting only p110α can block phosphoinositide 3-kinase signalling and tumour growth in certain cell types. Biochem. J. 438,53–62 (2011). 21668414 10.1042/BJ20110502 PMC3174055

[45] M. Fernández-Monreal, C. Sánchez-Castillo, J. A. Esteban,APPL1 gates long-term potentiation through its plekstrin homology domain. J. Cell Sci. 129,2793–2803 (2016). 27257087 10.1242/jcs.183475

[46] Y. Gutiérrez, S. López-García, A. Lario, S. Gutiérrez-Eisman, C. Delevoye, J. A. Esteban,KIF13A drives AMPA receptor synaptic delivery for long-term potentiation via endosomal remodeling. J. Cell Biol. 220,e202003183 (2021). 33999113 10.1083/jcb.202003183 PMC8129809

[47] A. Brachet, A. Lario, A. Fernández-Rodrigo, F. Heisler, Y. Gutiérrez, C. Lobo, M. Kneussel, J. Esteban,A kinesin 1-protrudin complex mediates AMPA receptor synaptic removal during long-term depression. Cell Rep. 36,109499 (2021). 34348158 10.1016/j.celrep.2021.109499

[48] A. K. Kraeuter, P. C. Guest, Z. Sarnyai,The Y-maze for assessment of spatial working and reference memory in mice. Methods Mol. Biol. 1916,105–111 (2019). 30535688 10.1007/978-1-4939-8994-2_10

[49] S. S. Moy, J. J. Nadler, A. Perez, R. P. Barbaro, J. M. Johns, T. R. Magnuson, J. Piven, J. N. Crawley,Sociability and preference for social novelty in five inbred strains: An approach to assess autistic-like behavior in mice. Genes Brain Behav. 3,287–302 (2004). 15344922 10.1111/j.1601-1848.2004.00076.x

[50] M. G. Butler, M. J. Dazouki, X.-P. Zhou, Z. Talebizadeh, M. Brown, T. N. Takahashi, J. H. Miles, C. H. Wang, R. Stratton, R. Pilarski, C. Eng,Subset of individuals with autism spectrum disorders and extreme macrocephaly associated with germline PTEN tumour suppressor gene mutations. J. Med. Genet. 42,318–321 (2005). 15805158 10.1136/jmg.2004.024646 PMC1736032

[51] H. O. Kalkman,The role of the phosphatidylinositide 3-kinase-protein kinase B pathway in schizophrenia. Pharmacol. Ther. 110,117–134 (2006). 16434104 10.1016/j.pharmthera.2005.10.014

[52] S. C. Borrie, H. Brems, E. Legius, C. Bagni,Cognitive dysfunctions in intellectual disabilities: The contributions of the Ras-MAPK and PI3K-AKT-mTOR pathways. Annu. Rev. Genomics Hum. Genet. 18,115–142 (2017). 28859574 10.1146/annurev-genom-091416-035332

[53] C. Gross, G. J. Bassell,Neuron-specific regulation of class I PI3K catalytic subunits and their dysfunction in brain disorders. Front. Mol. Neurosci. 7,12 (2014). 24592210 10.3389/fnmol.2014.00012 PMC3923137

[54] C. Gross, M. Nakamoto, X. Yao, C. B. Chan, S. Y. Yim, K. Ye, S. T. Warren, G. J. Bassell,Excess phosphoinositide 3-kinase subunit synthesis and activity as a novel therapeutic target in fragile X syndrome. J. Neurosci. 30,10624–10638 (2010). 20702695 10.1523/JNEUROSCI.0402-10.2010 PMC2924772

[55] C. Gross, N. Raj, G. Molinaro, A. G. Allen, A. J. Whyte, J. R. Gibson, K. M. Huber, S. L. Gourley, G. J. Bassell,Selective role of the catalytic PI3K subunit p110β in impaired higher order cognition in fragile X syndrome. Cell Rep. 11,681–688 (2015). 25921527 10.1016/j.celrep.2015.03.065 PMC4426038

[56] V. Y. Bolshakov, S. A. Siegelbaum,Postsynaptic induction and presynaptic expression of hippocampal long-term depression. Science 264,1148–1152 (1994). 7909958 10.1126/science.7909958

[57] S. H. R. Oliet, R. C. Malenka, R. A. Nicoll,Two distinct forms of long-term depression coexist in CA1 hippocampal pyramidal cells. Neuron 18,969–982 (1997). 9208864 10.1016/s0896-6273(00)80336-0

[58] S. S. Zakharenko, L. Zablow, S. A. Siegelbaum,Altered presynaptic vesicle release and cycling during mGluR-dependent LTD. Neuron 35,1099–1110 (2002). 12354399 10.1016/s0896-6273(02)00898-x

[59] A. M. Watabe, H. J. Carlisle, T. J. O’Dell,Postsynaptic induction and presynaptic expression of group 1 mGluR-dependent LTD in the hippocampal CA1 region. J. Neurophysiol. 87,1395–1403 (2002). 11877514 10.1152/jn.00723.2001

[60] Y. Tan, N. Hori, D. O. Carpenter,The mechanism of presynaptic long-term depression mediated by group I metabotropic glutamate receptors. Cell. Mol. Neurobiol. 23,187–203 (2003). 12735631 10.1023/A:1022949922364 PMC11530151

[61] M. Y. Xiao, Q. Zhou, R. A. Nicoll,Metabotropic glutamate receptor activation causes a rapid redistribution of AMPA receptors. Neuropharmacology 41,664–671 (2001). 11640920 10.1016/s0028-3908(01)00134-4

[62] E. M. Snyder, B. D. Philpot, K. M. Huber, X. Dong, J. R. Fallon, M. F. Bear,Internalization of ionotropic glutamate receptors in response to mGluR activation. Nat. Neurosci. 4,1079–1085 (2001). 11687813 10.1038/nn746

[63] M. W. Waung, B. E. Pfeiffer, E. D. Nosyreva, J. A. Ronesi, K. M. Huber,Rapid translation of Arc/Arg3.1 selectively mediates mGluR-dependent LTD through persistent increases in AMPAR endocytosis rate. Neuron 59,84–97 (2008). 18614031 10.1016/j.neuron.2008.05.014 PMC2580055

[64] E. D. Nosyreva, K. M. Huber,Developmental switch in synaptic mechanisms of hippocampal metabotropic glutamate receptor-dependent long-term depression. J. Neurosci. 25,2992–3001 (2005). 15772359 10.1523/JNEUROSCI.3652-04.2005 PMC6725134

[65] K. D. Micheva, R. W. Holz, S. J. Smith,Regulation of presynaptic phosphatidylinositol 4,5-biphosphate by neuronal activity. J. Cell Biol. 154,355–368 (2001). 11470824 10.1083/jcb.200102098 PMC2150764

[66] I. Milosevic, J. B. Sørensen, T. Lang, M. Krauss, G. Nagy, V. Haucke, R. Jahn, E. Neher,Plasmalemmal phosphatidylinositol-4,5-bisphosphate level regulates the releasable vesicle pool size in chromaffin cells. J. Neurosci. 25,2557–2565 (2005). 15758165 10.1523/JNEUROSCI.3761-04.2005 PMC6725155

[67] Z. Dou, M. Chattopadhyay, J. A. Pan, J. L. Guerriero, Y. P. Jiang, L. M. Ballou, Z. Yue, R. Z. Lin, W. X. Zong,The class IA phosphatidylinositol 3-kinase p110-β subunit is a positive regulator of autophagy. J. Cell Biol. 191,827–843 (2010). 21059846 10.1083/jcb.201006056 PMC2983054

[68] Z. Dou, J. A. Pan, H. A. Dbouk, L. M. Ballou, J. L. DeLeon, Y. Fan, J. S. Chen, Z. Liang, G. Li, J. M. Backer, R. Z. Lin, W. X. Zong,Class IA PI3K p110β subunit promotes autophagy through Rab5 small GTPase in response to growth factor limitation. Mol. Cell 50,29–42 (2013). 23434372 10.1016/j.molcel.2013.01.022 PMC3628298

[69] M. Kuijpers, G. Kochlamazashvili, A. Stumpf, D. Puchkov, A. Swaminathan, M. T. Lucht, E. Krause, T. Maritzen, D. Schmitz, V. Haucke,Neuronal autophagy regulates presynaptic neurotransmission by controlling the axonal endoplasmic reticulum. Neuron 109,299–313.e9 (2021). 33157003 10.1016/j.neuron.2020.10.005 PMC7837115

[70] C. Lüscher, K. M. Huber,Group 1 mGluR-dependent synaptic long-term depression: Mechanisms and implications for circuitry and disease. Neuron 65,445–459 (2010). 20188650 10.1016/j.neuron.2010.01.016 PMC2841961

[71] H. R. Monday, T. J. Younts, P. E. Castillo,Long-term plasticity of neurotransmitter release: Emerging mechanisms and contributions to brain function and disease. Annu. Rev. Neurosci. 41,299–322 (2018). 29709205 10.1146/annurev-neuro-080317-062155 PMC6238218

[72] M. Bosch, M. Sánchez-Álvarez, A. Fajardo, R. Kapetanovic, B. Steiner, F. Dutra, L. Moreira, J. A. López, R. Campo, M. Marí, F. Morales-Paytuví, O. Tort, A. Gubern, R. M. Templin, J. E. B. Curson, N. Martel, C. Català, F. Lozano, F. Tebar, C. Enrich, J. Vázquez, M. A. Del Pozo, M. J. Sweet, P. T. Bozza, S. P. Gross, R. G. Parton, A. Pol,Mammalian lipid droplets are innate immune hubs integrating cell metabolism and host defense. Science 370,eaay8085 (2020). 33060333 10.1126/science.aay8085

[73] S. Martínez-Bartolomé, P. Navarro, F. Martín-Maroto, D. López-Ferrer, A. Ramos-Fernández, M. Villar, J. P. García-Ruiz, J. Vázquez,Properties of average score distributions of SEQUEST: The probability ratio method. Mol. Cell. Proteomics 7,1135–1145 (2008). 18303013 10.1074/mcp.M700239-MCP200

[74] P. Navarro, J. Vazquez,A refined method to calculate false discovery rates for peptide identification using decoy databases. J. Proteome Res. 8,1792–1796 (2009). 19714873 10.1021/pr800362h

[75] E. Bonzon-Kulichenko, F. Garcia-Marques, M. Trevisan-Herraz, J. Vázquez,Revisiting peptide identification by high-accuracy mass spectrometry: Problems associated with the use of narrow mass precursor windows. J. Proteome Res. 14,700–710 (2015). 25494653 10.1021/pr5007284

[76] M. Trevisan-Herraz, N. Bagwan, F. García-Marqués, J. M. Rodriguez, I. Jorge, I. Ezkurdia, E. Bonzon-Kulichenko, J. Vázquez,SanXoT: A modular and versatile package for the quantitative analysis of high-throughput proteomics experiments. Bioinformatics 35,1594–1596 (2019). 30252043 10.1093/bioinformatics/bty815 PMC6499250

[77] P. Navarro, M. Trevisan-Herraz, E. Bonzon-Kulichenko, E. Núñez, P. Martínez-Acedo, D. Pérez-Hernández, I. Jorge, R. Mesa, E. Calvo, M. Carrascal, M. L. Hernáez, F. García, J. A. Bárcena, K. Ashman, J. Abian, C. Gil, J. M. Redondo, J. Vázquez,General statistical framework for quantitative proteomics by stable isotope labeling. J. Proteome Res. 13,1234–1247 (2014). 24512137 10.1021/pr4006958

[78] F. García-Marqués, M. Trevisan-Herraz, S. Martínez-Martínez, E. Camafeita, I. Jorge, J. A. Lopez, N. Méndez-Barbero, S. Méndez-Ferrer, M. A. Del Pozo, B. Ibáñez, V. Andrés, F. Sánchez-Madrid, J. M. Redondo, E. Bonzon-Kulichenko, J. Vázquez,A novel systems-biology algorithm for the analysis of coordinated protein responses using quantitative proteomics. Mol. Cell. Proteomics 15,1740–1760 (2016). 26893027 10.1074/mcp.M115.055905 PMC4858952

[79] J. Guillermet-Guibert, K. Bjorklof, A. Salpekar, C. Gonella, F. Ramadani, A. Bilancio, S. Meek, A. J. H. Smith, K. Okkenhaug, B. Vanhaesebroeck,The p110β isoform of phosphoinositide 3-kinase signals downstream of G protein-coupled receptors and is functionally redundant with p110γ. Proc. Natl. Acad. Sci. U.S.A. 105,8292–8297 (2008). 18544649 10.1073/pnas.0707761105 PMC2448830

[80] A. A. Walf, C. A. Frye,The use of the elevated plus maze as an assay of anxiety-related behavior in rodents. Nat. Protoc. 2,322–328 (2007). 17406592 10.1038/nprot.2007.44 PMC3623971

