# Supplementary material for: Functional specialization of different PI3K isoforms for the control of neuronal architecture, synaptic plasticity, and cognition
Source: Sci Adv. 2022 Nov 23;8(47):eabq8109. doi: 10.1126/sciadv.abq8109 (PMC9683729; doi:10.1126/sciadv.abq8109)
Supplement: Supplementary file 1 — Figs. S1 to S11 [file sciadv.abq8109_sm.pdf]

Science Advances
AAAS

Supplementary Materials for

Functional specialization of different PI3K isoforms for the control of neuronal architecture, synaptic plasticity, and cognition

Carla Sánchez-Castillo *et al.*

Corresponding author: José A. Esteban, jaesteban@cbm.csic.es

Sci. Adv. **8**, eabq8109 (2022)
DOI: 10.1126/sciadv.abq8109

The PDF file includes:

Figs. S1 to S11
Legend for data S1

Other Supplementary Material for this manuscript includes the following:

Data S1

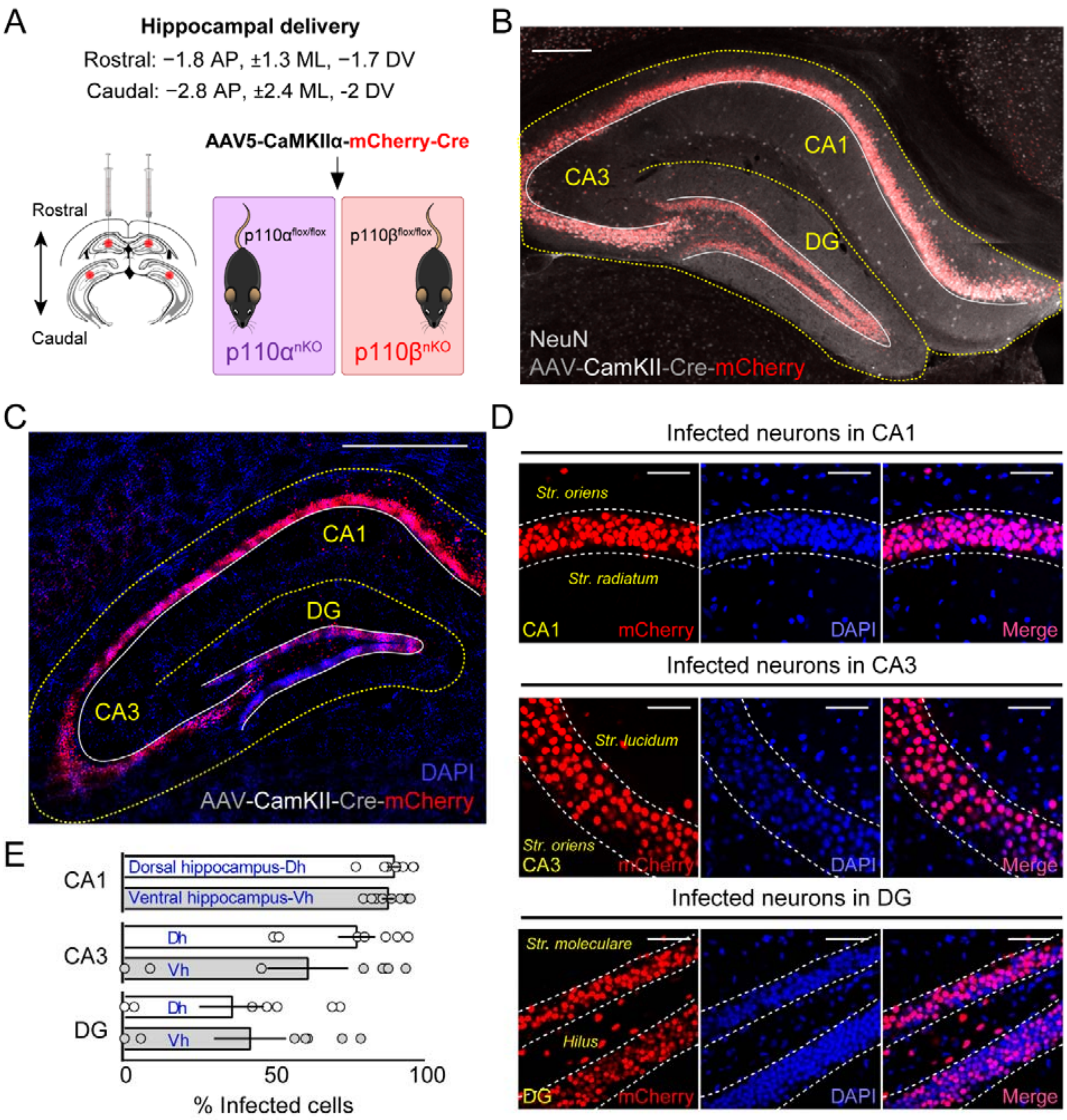

Figure S1. In vivo stereotaxic AAV injections effectively mediate CamKII-driven expression of Cre recombinase on neuronal layers of the hipocampus. (A) Experimental design for the ablation of p110α and p110β in hippocampal neurons from p110α$^{flox/flox}$ and p110β$^{flox/flox}$ mice for the generation of p110α^{nKO} and p110β^{nKO}. **(B)** Merged representative image of mCherry-Cre expression (shown in red) in the hippocampus one month after AAV infection in combination with the neuronal marker NeuN (shown in grey). Scale bar = 100 µm. **(C)** Merged representative image of viral infection in a sagittal slice of mouse hippocampus. White lines underline pyramidal neuron layer and granule cell layer on the dentate gyrus (DG). Dotted yellow lines delimitate pyramidal neuron layer and granule cell layer on the DG. **(D)** High magnification pictures from CA1, CA3

and DG subregions (top to bottom). From right to left: AAV infected neurons labeled by mCherry (shown in red), DAPI nuclear stain (shown in blue) and merged images. **(E)** Quantification of the number of infected cells (mCherry labeled nuclei) *versus* total number of cells (DAPI nuclear staining) on the pyramidal CA1 and CA3 and the granular DG neuron layers. The percentage of infected neurons was quantified separately on the rostral and caudal zones of the hippocampus in order to see the targeting efficiency of the different subregions along the rostro-caudal axis. N= 4 infected mice with bilateral AAV-Cre injections. A total of 16 slices were quantified per mice (8 from dorsal and 8 from ventral hippocampus).

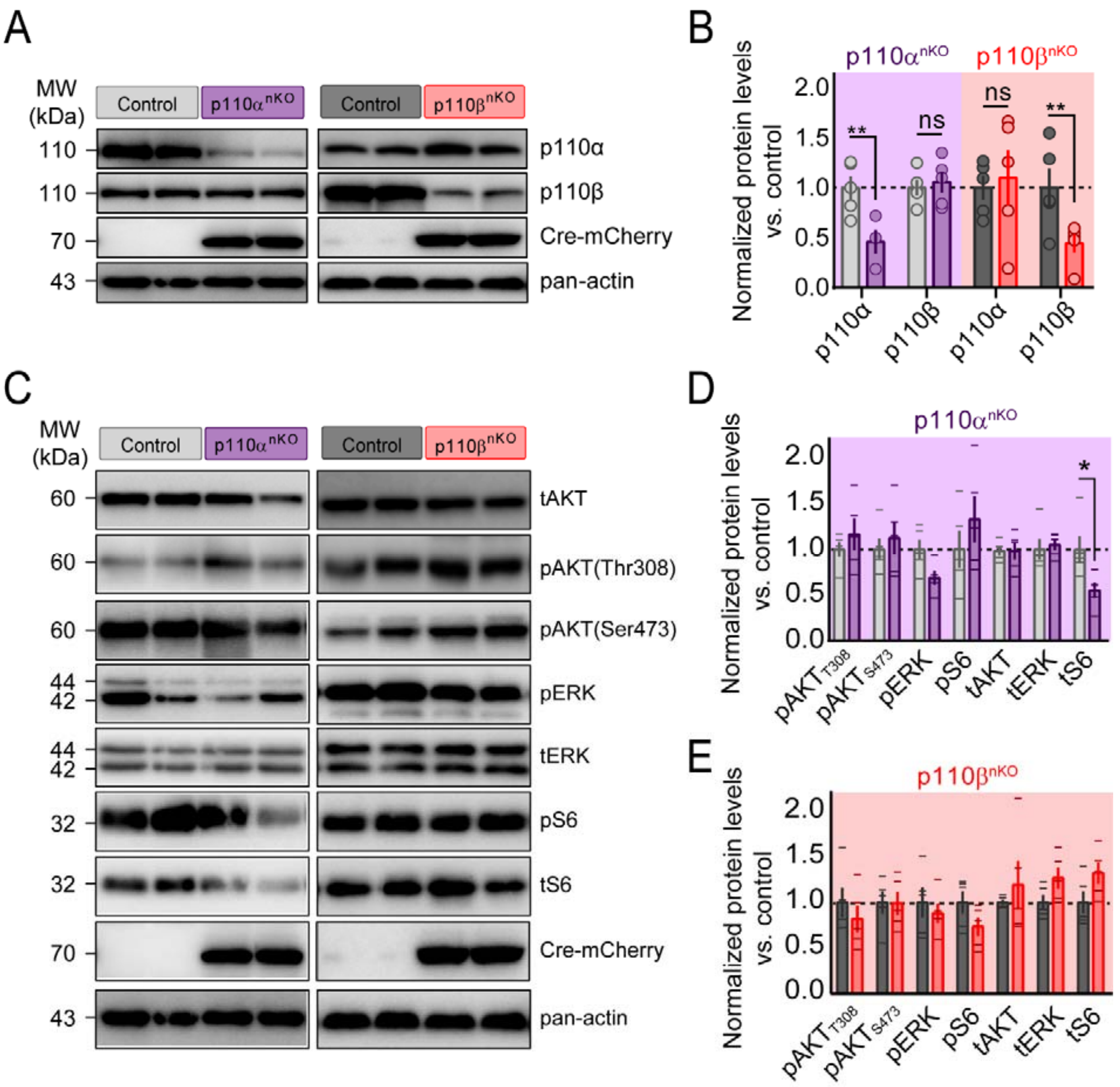

Figure S2. Cre-LoxP mediated conditional knockout of p110α and p110β on CA1 pyramidal neurons is selective and effective. (A) Representative western blots of whole extracts of *in vivo* infected hippocampi with AAV driving the expression of Cre-mCherry on neurons. Membranes were probed for p110α, p110β, mCherry (infection level control) and pan-actin as loading control. **(B)** Quantification of the protein levels for p110α and p110β on p110α^{nKO} and p110β^{nKO} mice and their respective controls. Protein levels were normalized to actin. Data are displayed as the percentage versus the control group. Statistical differences were assessed by Wilcoxon matched-pairs signed rank test. **p<0.01: p110α^{nKO} vs. control (p=0.004) and p110β^{nKO} vs. control (p=0.004). ns: not significant. n=5 mice/condition. **(C)** Representative western blots of whole extracts from p110α^{nKO} and p110β^{nKO} mice and their respective controls for assessing the expression of downstream targets of the PI3K pathway. Membranes were probed for phospho-AKT (in both T308 and S473 residues), total AKT (tAKT), phosho-ERK (pERK) and total ERK (tERK) and phospho-S6 (pS6) and total S6 (tS6). mCherry was used for checking the infection

levels and actin as loading control. **(D, E)** Quantification of phosphorylation and total protein levels from western blots as the ones shown in (C). For pAKT, pERK and pS6, data were normalized by their respective total levels. For tAKT, tERK and tS6, protein levels were normalized to actin. Data are displayed as the percentage *versus* the control group. No statistical differences were detected by Wilcoxon matched-pairs signed rank test except for that observed in total S6 level upon p110α deletion (* $p<0.05$: p110α^{nKO} vs. control). n=5 mice per condition. Bars represent mean±SEM.

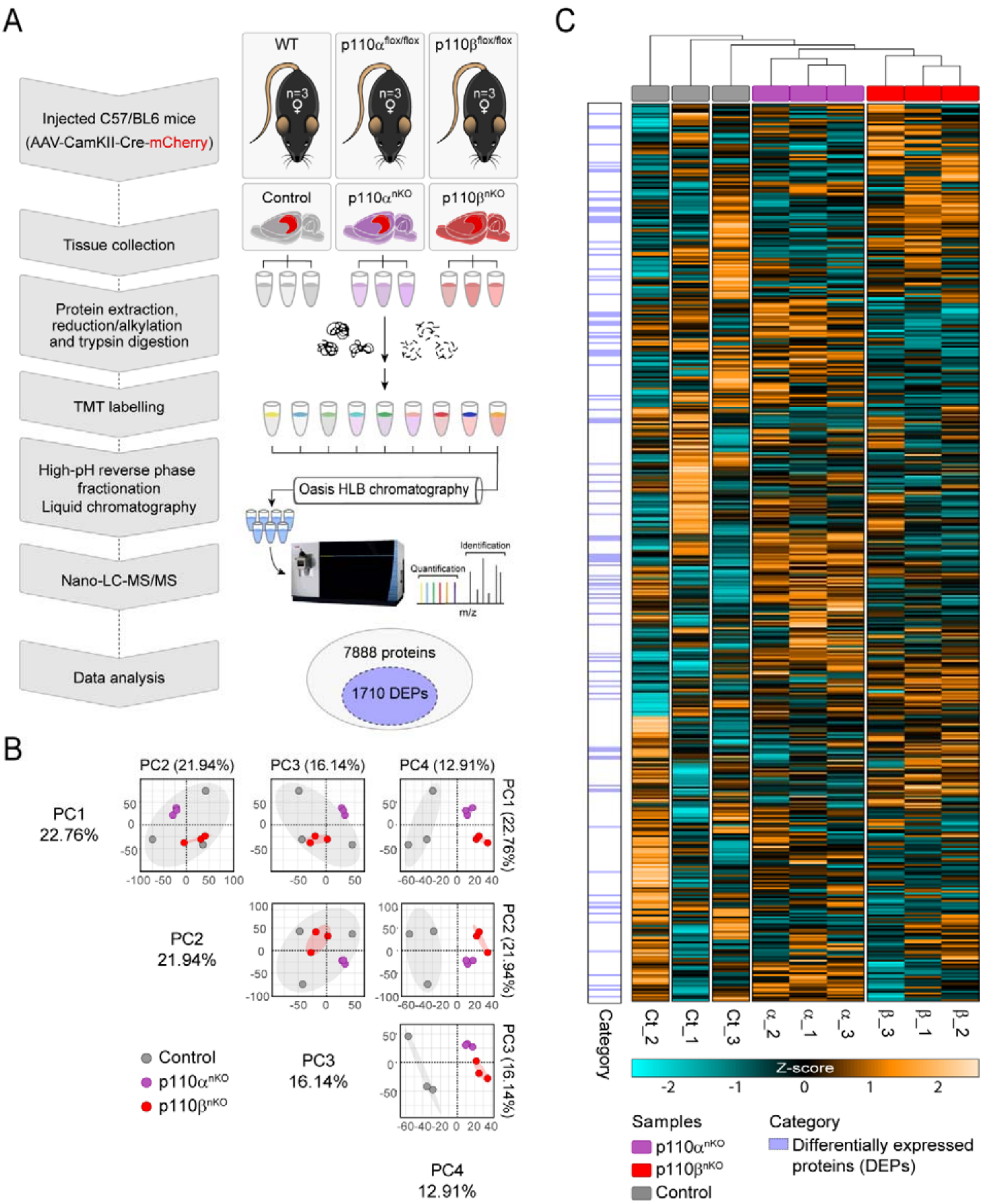

Figure S3. Proteomic analysis of whole hippocampal extracts from p110α^{nKO} and p110β^{nKO} mice. (A) Experimental workflow of the proteomic analysis of hippocampal extracts from p110α^{nKO} and p110β^{nKO} mice and their respective controls. 1710 differentially expressed proteins

were identified among 7888 total proteins (|Zq|>1.2 and p value<0.05). **(B)** Principal component analysis (PCA) of the proteomic data covering 73% of the dataset variance. PC1-4 are the eigenvectors of principal components. The fraction of the total variance stored in each PC is also represented. **(C)** Clustered heat map showing the expression patterns of the 7888 total identified proteins (rows) for our 9 samples (columns). Unsupervised hierarchical clustering based on Euclidean distances was performed for both rows and columns and data was scaled by rows for representation (z-scores). The color scale shows standard deviations from the mean of the row, with cyan for downregulation and orange for upregulated protein levels. The left annotation panel depicts in blue the position of the 1710 differentially expressed proteins. n=3 mice per condition.

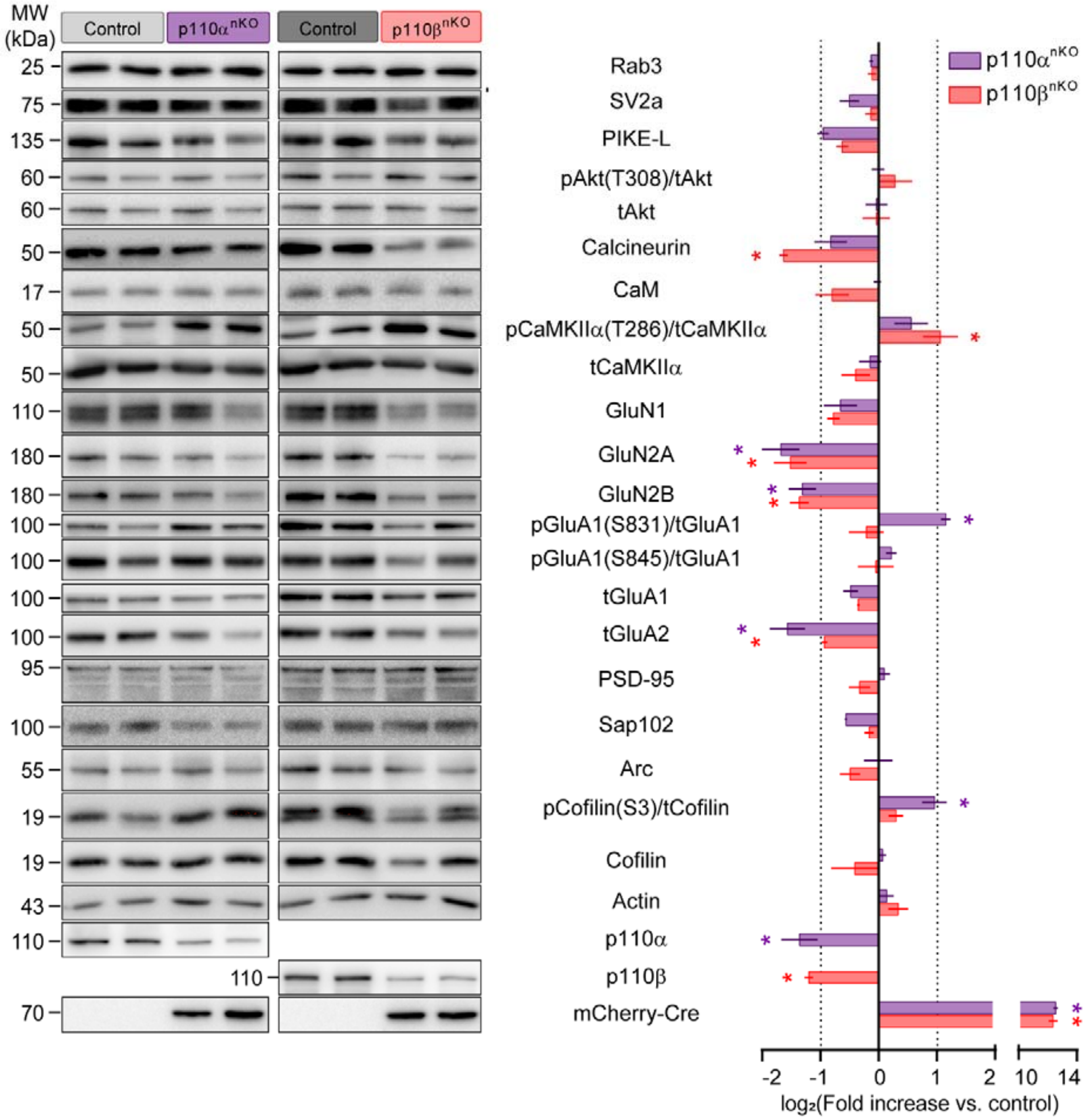

Figure S4. Analysis of proteomic targets by western blot reinforces non-overlapping roles for both isoforms. Characterization by western blot and quantification of different proteins related to postsynaptic and presynaptic function. Representative western blots of whole extracts of *in vivo* infected hippocampi with AAV-CamkII-Cre are displayed in the left. Bar graphs for the quantification of different protein in p110αnKO and p110βnKO hippocampus and their respective controls are shown on the right. Expression changes are quantified as $\log_2$(fold change) *versus* the control group. Data are displayed as mean±SEM. Statistical differences were assessed by Wilcoxon matched-pairs signed rank test (*p<0.05, **p<0.01: p110α^{nKO} vs. control and p110β^{nKO} vs. control; ns: not significant). n=5 mice per condition.

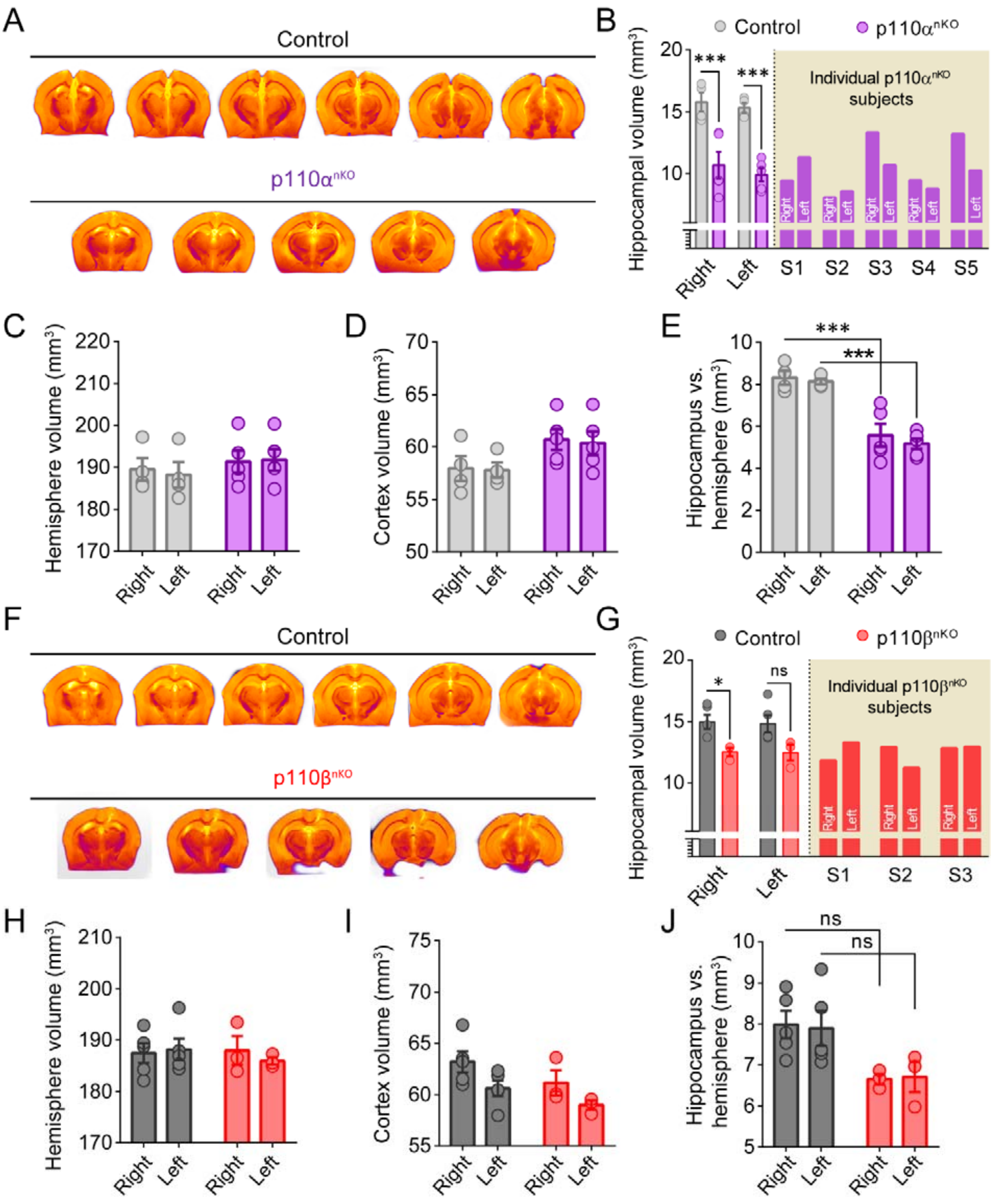

Figure S5. Differential contribution of p110α and p110β for maintaining proper hippocampal size. *Ex vivo* MRI analysis of the brain from p110α^{nKO} and p110β^{nKO} mice one month after AAV-CamKII-Cre hippocampal infection. **(A)** Representative coronal sections displaying the hippocampus along the rostro-caudal axis in control (upper panel) and in p110α^{nKO}

(lower panel) mice. **(B)** Hippocampal volume quantification for the right and the left hemisphere in both p110α^{nKO} and control mice. The graph shows the mean right and left hippocampal volume for each experimental condition and individual hippocampal volumes for each p110α^{nKO} mice. Two-way repeated measures ANOVA demonstrated a significant effect for AAV infection in p110α$^{flox/flox}$ mice (($F(1;7)= 30.24$); $p=0.0009$); Bonferroni's post-test (***$p<0.005$ vs vehicle for p110α^{nKO}). **(C, D)** Quantification of the volume for the right and the left hemisphere (C) and the cortex (D) in p110α^{nKO} and control mice. No differences were detected by two-way ANOVA. **(E)** Quantification of the right and left hippocampal volume normalized by the total hemisphere volume for p110α^{nKO} and control mice. Two-way ANOVA demonstrated a significant effect for AAV infection ($F(1;14)= 57.42$); $p<0.0001$); Tukey's post-test ***$p<0.005$ vs vehicle. **(F)** Representative coronal sections along the rostro caudal axis displaying the hippocampal formation in control (upper panel) and in p110β^{nKO} mice (lower panel). **(G)** Hippocampal volume quantification for the right and the left hemisphere in both p110β^{nKO} and control mice. The graph shows the mean right and left hippocampal volume for each experimental condition and individual hippocampal volumes for each p110β^{nKO} mice. Two-way repeated measures ANOVA demonstrated a significant effect for AAV infection in p110β$^{flox/flox}$ mice ($F(1;6)= 8.205$); $p=0.0286$); Bonferroni's post-test *$p<0.05$ vs vehicle for p110β^{nKO}). **(H, I)** Quantification of the volume for the right and the left hemisphere (H) and the cortex (I) in p110β^{nKO} and control mice. No differences were detected by two-way repeated measures ANOVA. **(J)** Quantification of the right and left hippocampal volume normalized by the total hemisphere volume for p110β^{nKO} and control mice. Two-way repeated measures ANOVA demonstrated a significant effect for AAV infection ($F(1;12)= 10.36$); $p=0.0071$); Tukey's post-test ns: not significant vs vehicle. p110α$^{flox/flox}$ (vehicle, n=4 and p110α^{nKO}, n=5) and p110β$^{flox/flox}$ mice (vehicle, n=5 and p110β^{nKO}, n=3). Data are displayed as Mean±SEM.

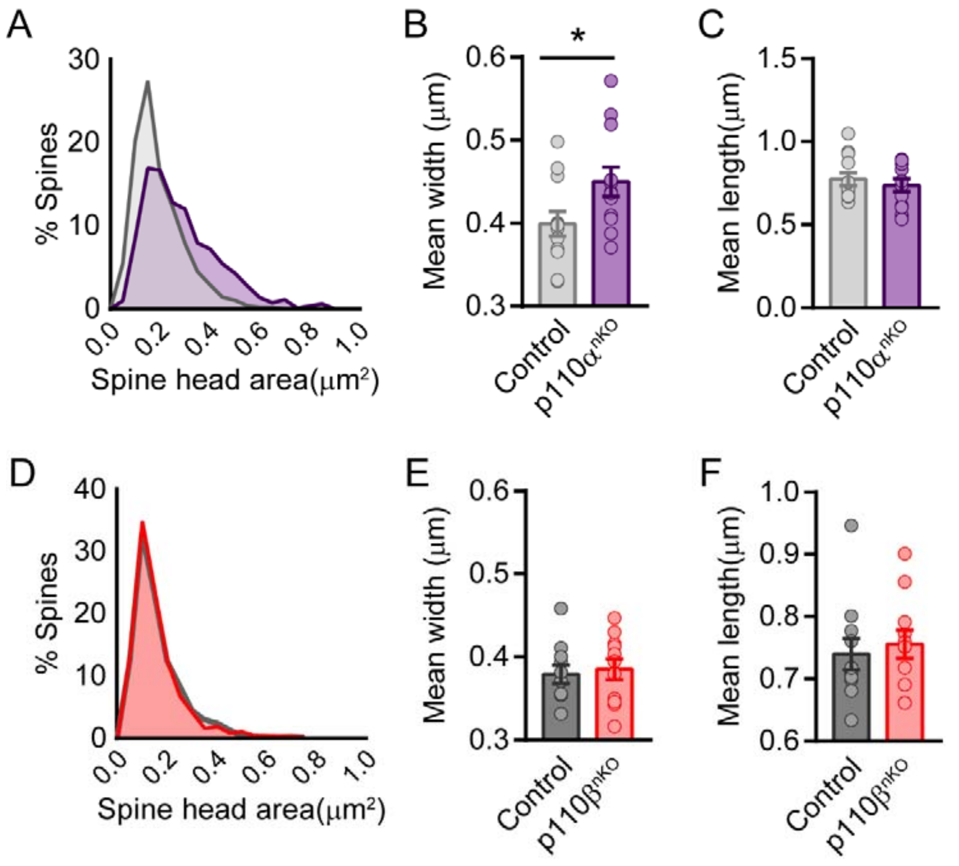

Figure S6. Differential contribution of p110α and p110β for neuronal and spine morphology. **(A)** Frequency histogram of the spine head area of p110α knockout neurons and their respective controls. Spine head area was measured in GFP neurons 24 hours after infection. Vehicle control: n=1422; p110α^{nKO}: n=792 spines from 3 mice/condition. **(B, C)** Quantification of the mean width and length of GFP positive spines from p110α knockout neurons and their respective controls. Data were compared by Mann-Whitney (*$p<0.05$ vs. control neurons). Vehicle control: n=11; p110α^{nKO}: n=12 dendrites from 3 mice/condition. Data are displayed as the spine average per dendrite. **(D)** Similar to (A), from p110β knockout neurons. Vehicle control: n= 1503; p110β^{nKO}: n=1457 spines from 3 mice/condition. **(E, F)** Similar to (B, C) from p110β knockout mice. Vehicle control: n= 11; p110β^{nKO}: n=12 dendrites from 3 mice/condition.

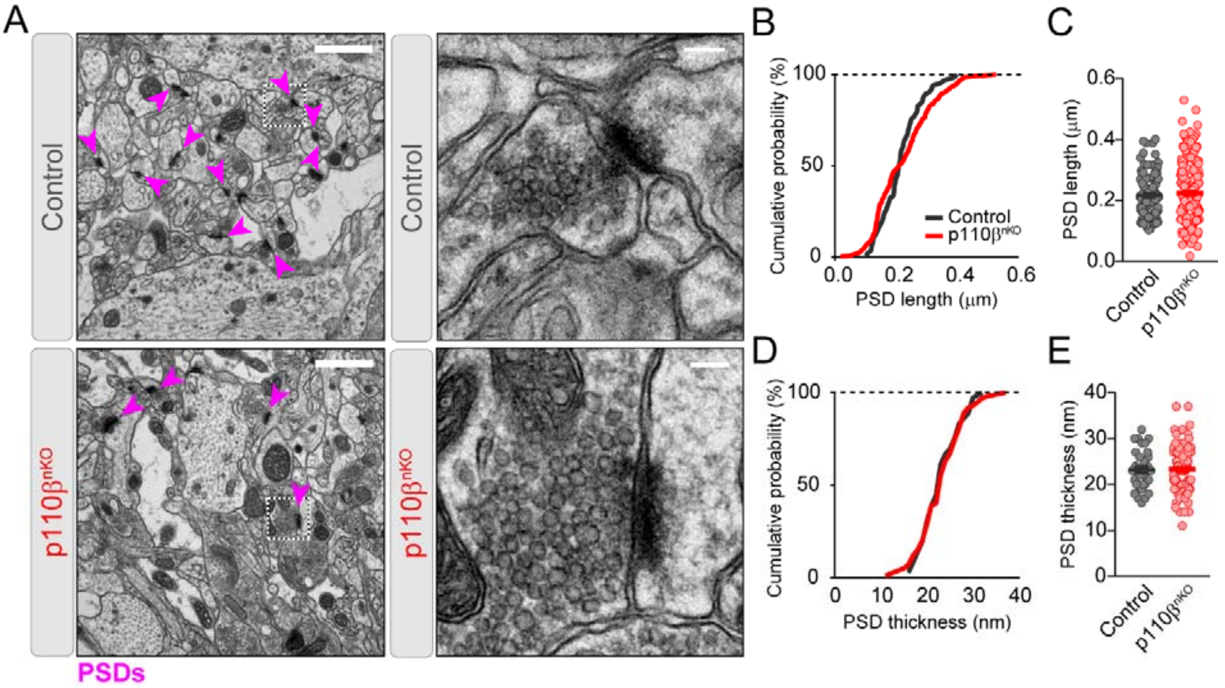

Figure S7. PSD thickness and length remained normal in p110β^{nKO} mice. (A) Representative electron micrograph examples from CA1 *stratum radiatum* of saline (control, upper panels) and AAV (p110β^{nKO}, lower panels) injected p110β$^{flox/flox}$ mice. Right panels depict higher magnification pictures from representative synapses. Scale bar= 1 μm. **(B-E)** Cumulative frequency distributions (B, D) and scatter plots graphs (C, E) for PSD length (B, C) and PSD thickness (D, E). No differences were found by Mann-Whitney test. PSD length: Vehicle, n= 219; p110β^{nKO}, n=287. PSD thickness: Vehicle, n= 213; p110β^{nKO}, n=283 synapses from 3 mice per condition. Data are displayed as mean±SEM.

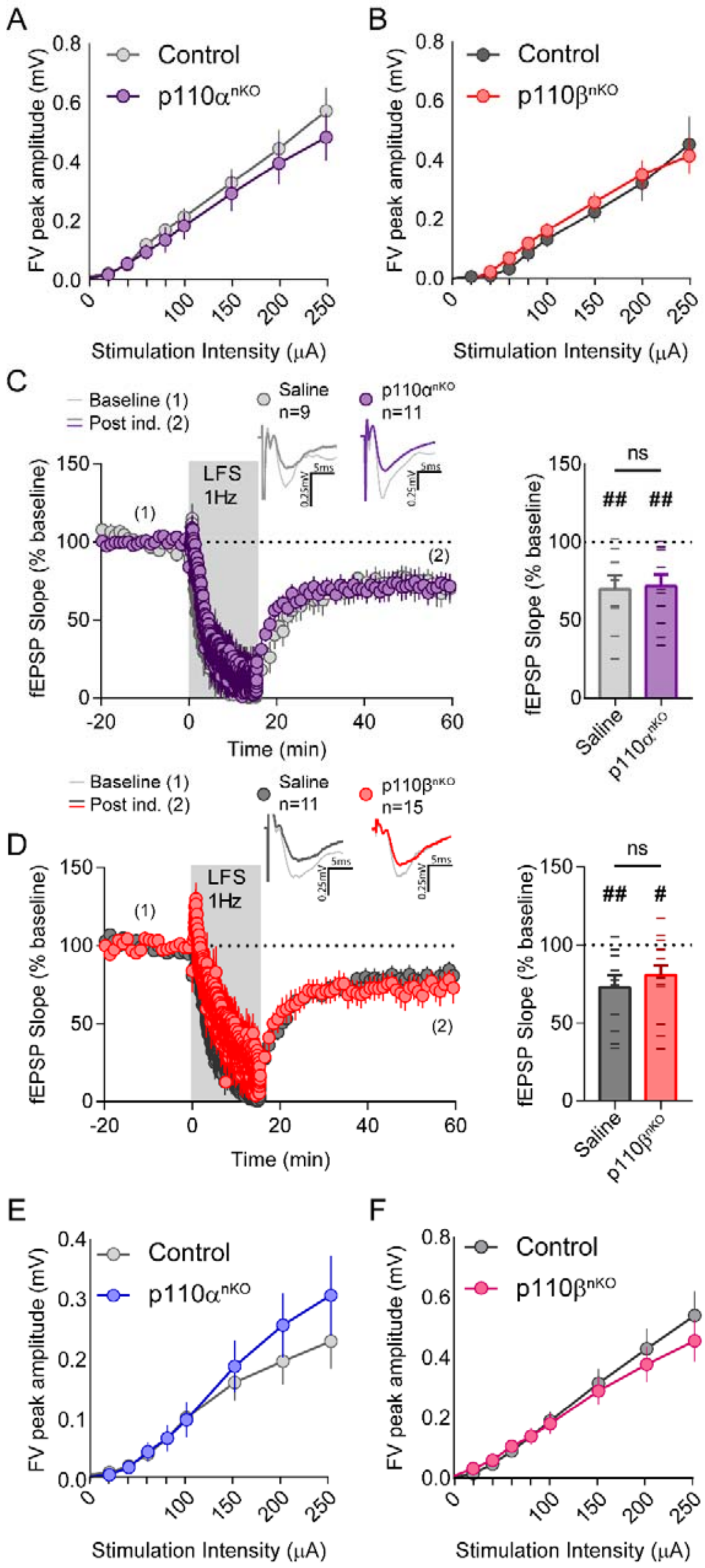

A
Control
p110α^nKO
FV peak amplitude (mV)
Stimulation Intensity (μA)
B
Control
p110β^nKO
FV peak amplitude (mV)
Stimulation Intensity (μA)
C
Baseline (1)
Post ind. (2)
Saline n=9
p110α^nKO n=11
LFS 1Hz
(1)
(2)
fEPSP Slope (% baseline)
Time (min)
ns
##
Saline
p110α^nKO
Baseline (1)
Post ind. (2)
Saline n=11
p110β^nKO n=15
D
LFS 1Hz
(1)
(2)
fEPSP Slope (% baseline)
Time (min)
ns
#
Saline
p110β^nKO
E
Control
p110α^nKO
FV peak amplitude (mV)
Stimulation Intensity (μA)
F
Control
p110β^nKO
FV peak amplitude (mV)
Stimulation Intensity (μA)

Figure S8. Fiber volley amplitude and NMDA receptor-dependent LTD in p110α^{nKO} and p110β^{nKO} mice. Fiber volley response over increasing stimulation intensities (from 20 to 250 μA) during input–output curve measures from acute hippocampal slices from p110α **(A)** or p110β **(B)** neuronal knockout mice and their controls. No differences were detected by two-way repeated measures ANOVA. p110α$^{flox/flox}$: vehicle, n=13; p110α^{nKO}, n=12 slices. p110β$^{flox/flox}$: vehicle, n=19; p110β^{nKO}, n=16 slices. More than 4 mice per condition. (**C, D**) Time course of NMDA receptor-dependent LTD induced by 900 pulses at 1 Hz in the presence of picotroxin in p110α^{nKO} (**C**) and p110β^{nKO} slices (**D**) and their respective control mice. Right, bar graphs showing the average fEPSP slope from the last 5min of recording. Wilcoxon statistical test was used to evaluate LTD expression with respect to baseline (#P < 0.05; ##P <0.01). No Statistical differences were found between conditions by Mann-Whitney test. p110α$^{fl/fl}$: vehicle, n=8 and p110α^{nKO}, n=10 slices. p110β$^{fl/fl}$: vehicle, n=10 and p110β^{nKO}, n=12 slices from 4 mice/condition. Representative traces are shown above the graphs. **(E, F)** Similar experiments from p110α (**E**) and p110β (**F**) floxed mice and their controls infected with AAV CaMKII-Cre in the CA1 hippocampal region. No differences were detected by two-way repeated measures ANOVA. p110α$^{flox/flox}$: vehicle, n=11; p110α^{nKO}=14 slices. 4 mice. p110β$^{flox/flox}$: vehicle, n=30; p110β^{nKO}=17 slices. More than 5 mice. For all panels, data are displayed as mean±SEM.

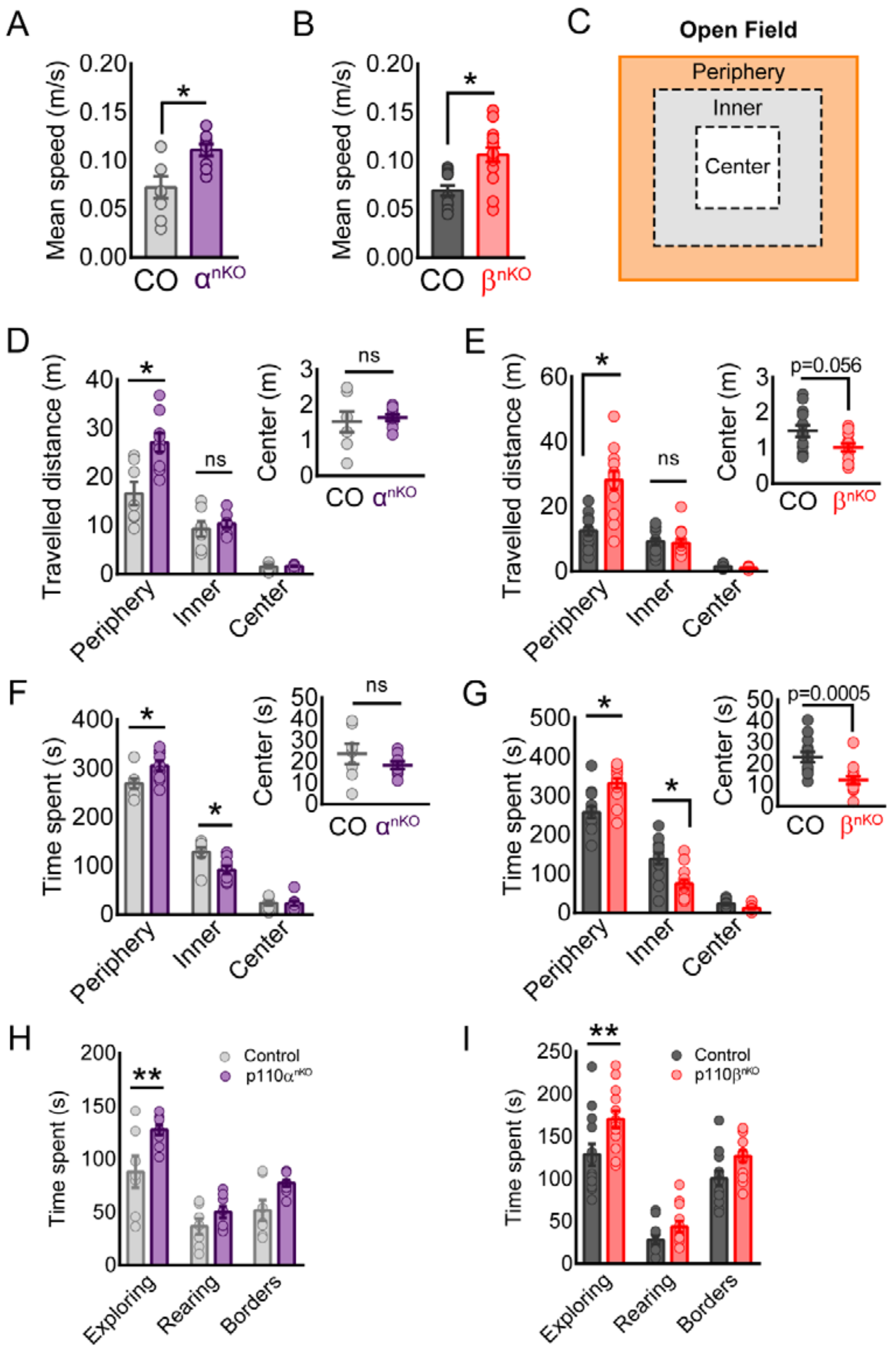

Figure S9. Locomotion activity of p110α^{nKO} and p110β^{nKO} in the open field arena. (A, B) Quantification of the mean average speed for the entire duration of the open field test for p110α (A) and p110β (B) infected mice and their respective controls. Data were compared by Mann-Whitney test (*p<0.05 vs control mice). p110α$^{flox/flox}$: vehicle, n=8; p110α^{nKO}, n=9. p110β$^{flox/flox}$:

vehicle, n=13; p110β^{nKO}, n=15. Data are displayed as mean±SEM. **(C)** Division of zones in the Open Field Arena for evaluation of anxiety-like behavior. **(D, E)** Quantification of the mean travelled distance for the entire duration of the test for p110α (D) and p110β (E) floxed mice. These quantifications were performed separately for the periphery, inner and center zones of the arena. Travelled distance in the center is shown in the inset dot plot graph. **(F, G)** Quantification of the time mice spent on each region of the arena for p110α (F) and p110β (G) floxed mice. Time spent in the center is shown in the inset dot plot graph. Statistical analysis was performed with a two-way repeated-measures ANOVA and Bonferroni post hoc test (*p<0.05 vs. control mice). p110α$^{flox/flox}$: vehicle, n=8; p110α^{nKO}, n=9. p110β$^{flox/flox}$: vehicle, n=13; p110β^{nKO}, n=15. **(H, I)** Quantification of the time spent exploring (total, rearing and borders exploration) during the open field test displayed by p110α^{nKO} (H) and p110β^{nKO} (I). Data were compared by two-way repeat measures ANOVA and Bonferroni post-test (**p<0.01 vs control). p110α$^{flox/flox}$: vehicle, n=7; p110α^{nKO}, n=9. p110β$^{flox/flox}$: vehicle, n=13; p110β^{nKO}, n=13.

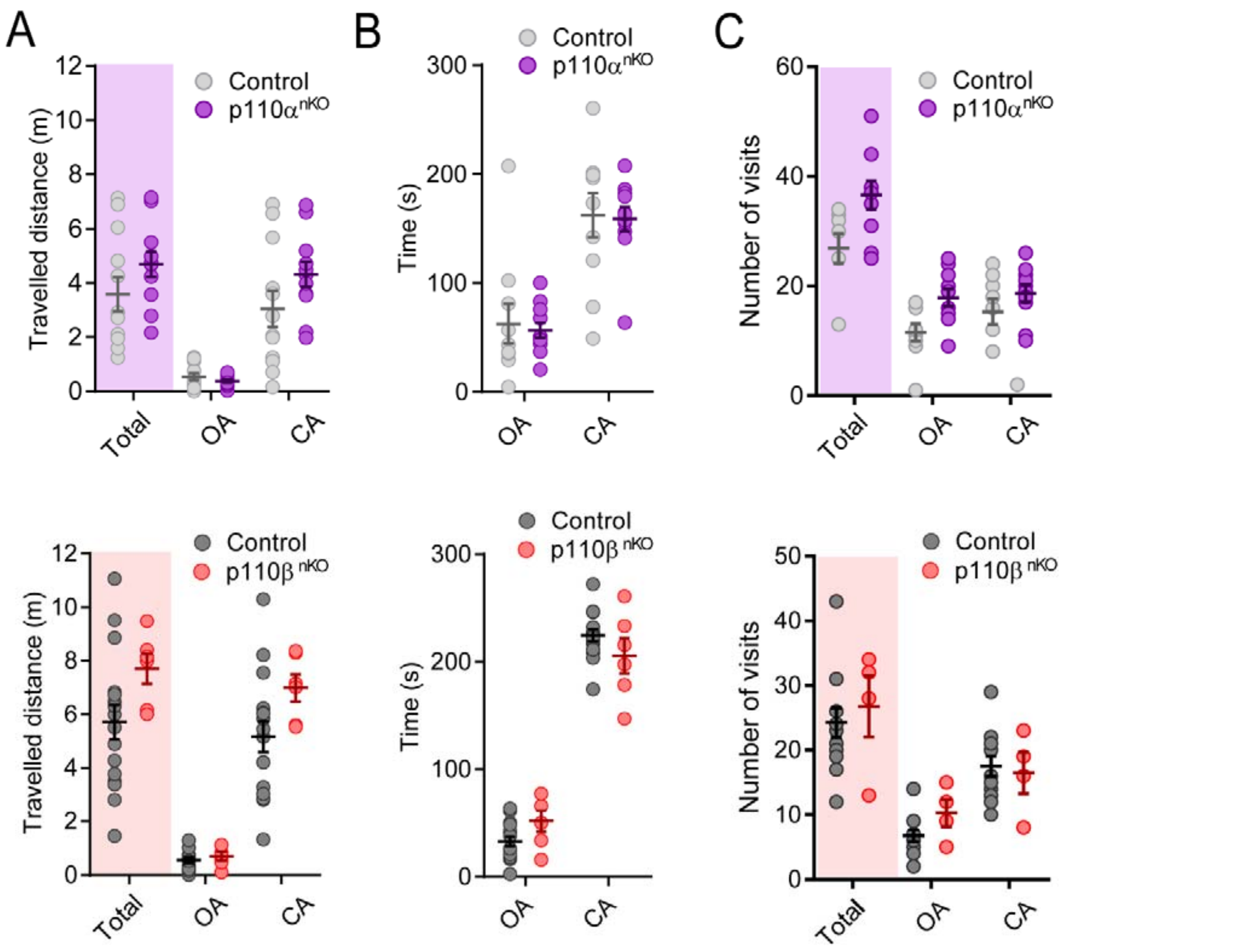

Figure S10. Anxiety-like behavior of p110α^{nKO} and p110β^{nKO} in the elevated plus maze. Quantification of the total travelled distance **(A)**, the average time spent **(B)** and the number of visits **(C)** in the Open (OA) and Closed Arms (CA) of the Elevated Plus Maze for p110α^{nKO} (upper panels) and p110β^{nKO} (lower panels) mice along the 5 min of the test. Total values (OA+CA) are also shown. In all cases, individual values are represented along with the mean±SEM for each condition. p110α$^{flox/flox}$: vehicle, n=8; p110α^{nKO}, n=10. p110β$^{flox/flox}$: vehicle, n=16; p110β^{nKO}, n=6. No differences were detected by two-way repeated-measures ANOVA.

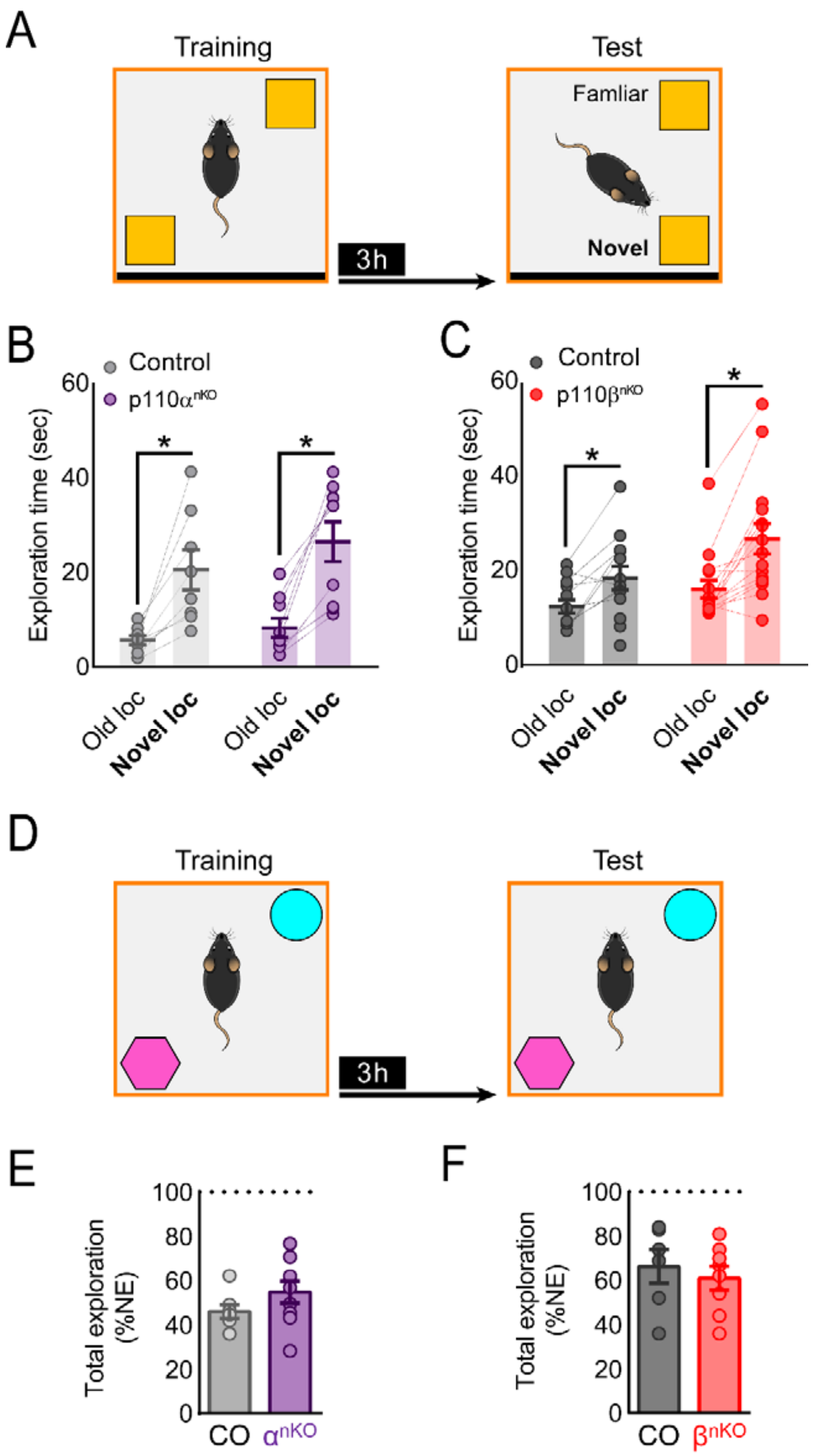

Figure S11. Lack of p110α or p110β on hippocampal neurons does not alter spatial memory in the novel object location (NOL). (A) Standard experimental scheme of the novel object location (NOL) recognition task. In the training phase mice are allowed to explore two identical

objects in an open field arena with spatial cues (black wall). After a 3 h interval mice are subjected to the testing phase in which they are exposed again to both objects, but one has been displaced. **(B, C)** Quantification of the time spent exploring the objects in the novel and the old location for p110α^{nKO} and p110β^{nKO}. Statistical analysis: old vs. novel location for controls, p110α^{nKO} or p110β^{nKO} was performed with two-way repeated-measures ANOVA and Bonferroni post-hoc test (*p<0.05 vs. old location). p110α$^{flox/flox}$ (vehicle, n=8 and p110α^{nKO}, n=8) and p110β$^{flox/flox}$ mice (vehicle, n=10 and p110β^{nKO}, n=15). (**D**) Experimental model of the object recognition task for the re-exposure testing. After habituating in an empty box on day 1, mice were exposed to two novel objects on the following day (training), and then re-exposed to the same objects 3 h later (re-exposure). (**E, F**) During re-exposure, p110α^{nKO} (**E**) and p110β^{nKO} (**F**) spent the same time exploring the objects as control saline mice. Two-tailed Mann-Whitney test demonstrated no differences between saline and infected mice. p110α$^{fl/fl}$: vehicle, n=7 and p110α^{nKO}, n=8. p110β$^{fl/fl}$: vehicle, n=6 and p110β^{nKO}, n=8.NE, novel exploration. For all panels, data are displayed as mean±SEM.

Data S1. Complete dataset and statistical analysis for p110α^{nKO}, p110β^{nKO} proteomic comparison.
